# Multidisciplinary providers’ perspectives on timely predialysis arteriovenous access preparation: An exploratory mixed-methods study in upper Northern Thailand

**DOI:** 10.1371/journal.pone.0359863

**Published:** 2026-10-05

**Authors:** Amaraporn Rerkasem, Kochaphan Phirom, Pak Thaichana, Prit Kusirisin, Puntapong Taruangsri, Suwinai Saengyo, Sasinat Pongtam, Wanicha Pungchompoo, Kittipan Rerkasem

**Affiliations:** 1 Research Institute for Health Sciences, Chiang Mai University, Chiang Mai, Thailand; 2 School of Health Sciences Research, Research Institute for Health Sciences (RIHES), Chiang Mai University, Chiang Mai, Thailand; 3 Division of Nephrology, Department of Internal Medicine, Faculty of Medicine, Chiang Mai University, Chiang Mai, Thailand; 4 Department of Internal Medicine, Nakornping Hospital, Chiang Mai, Thailand; 5 Faculty of Nursing, Chiang Mai University, Chiang Mai, Thailand; 6 Department of Surgery, Faculty of Medicine, Maharaj Nakorn Chiang Mai Hospital, Chiang Mai University, Chiang Mai, Thailand; 7 Environmental and Occupational Health Sciences and Non-Communicable Diseases Research Center, Chiang Mai University, Chiang Mai, Thailand; 8 Clinical Surgical Research Center, Department of Surgery, Faculty of Medicine, Chiang Mai University, Chiang Mai, Thailand; Chulalongkorn University Faculty of Medicine, THAILAND

## Abstract

**Background:**

Timely creation of arteriovenous (AV) access before hemodialysis initiation is recommended to reduce catheter-related complications and improve clinical outcomes. However, predialysis AV access preparation remains suboptimal in Thailand despite guideline recommendations and expanded hemodialysis services. Evidence comparing perspectives among professional groups involved in the AV access care pathway is limited. This exploratory regional study compared the perspectives of nephrologists, vascular surgeons, and dialysis nurses in Upper Northern Thailand and identified supportive strategies that providers considered relevant to improving timely AV access preparation.

**Methods:**

We conducted a cross-sectional convergent mixed-methods study using a self-administered survey among nephrologists, vascular surgeons, and dialysis nurses practicing in hospitals and dialysis clinics across Upper Northern Thailand from February to April 2025. Likert-scale responses were summarized and compared across professional groups, and brief open-ended responses were analyzed thematically. Quantitative and qualitative findings were integrated during interpretation.

**Results:**

A total of 113 providers participated, including 78 nurses, 19 surgeons, and 16 nephrologists. Integration of the quantitative and qualitative findings identified five priority barriers: limited patient understanding, late presentation, procedural waiting times, insurance or reimbursement constraints, and clinical suitability concerns. Although the broad barrier patterns were shared, the emphasis placed on particular barriers varied across professional groups. Four supportive strategies were consistently endorsed: predictable financial coverage and reimbursement mechanisms, a dedicated vascular access coordinator, electronic referral and longitudinal follow-up systems, and proactive, stage-aligned AV access planning guidelines. Qualitative responses provided context by showing that providers associated these shared strategies with different role-related considerations, including patient preparation, referral coordination, procedural feasibility, and timing of care.

**Conclusions:**

Providers in this regional study identified shared priorities for improving timely predialysis AV access preparation, while differing in the meanings and considerations they associated with those priorities. These context-specific, hypothesis-generating findings may inform locally adapted service improvement and future evaluation of the proposed strategies.

## Introduction

### The global challenge of predialysis arteriovenous access preparation

End-stage kidney disease (ESKD) affects millions of people worldwide, and hemodialysis remains the predominant modality of kidney replacement therapy (KRT). International clinical guidelines emphasize early and individualized planning for arteriovenous (AV) access—including arteriovenous fistulas (AVFs) and arteriovenous grafts (AVGs)—before hemodialysis initiation [1]. Timely AV access preparation is associated with lower catheter dependence and reduced risk of infection, hospitalization, and mortality [2]. Nevertheless, timely predialysis AV access preparation remains suboptimal in many settings [2], and substantial numbers of patients continue to initiate hemodialysis using central venous catheters.

### Thailand’s ESKD burden and evolving policy context

Thailand has one of the highest reported incidences of ESKD worldwide [3] and has achieved near-universal access to dialysis through the Universal Coverage Scheme, Social Security System, and Civil Servant Medical Benefit Scheme [4]. In 2022, the longstanding “Peritoneal Dialysis First” policy was replaced by a “free choice” model, expanding access to hemodialysis, including full reimbursement under the Universal Coverage Scheme. However, expansion of hemodialysis services has not been accompanied by a corresponding increase in timely predialysis AV access preparation [4]. Unplanned initiation with central venous catheters therefore remains common and is associated with adverse early outcomes and additional health system burden [5]. Providers’ perspectives on barriers and supportive factors within this changing regional context remain incompletely understood.

### Gaps in understanding provider perspectives

Previous studies have described patient-level barriers to timely AV access creation, including fear, denial, financial concerns, and social influences, as well as organizational constraints involving predialysis care pathways and communication [6–8]. However, evidence comparing the perspectives of professional groups directly involved in AV access preparation remains limited. Existing studies have often examined barriers at the dialysis- or nephrology-service level while combining different healthcare professionals into a single provider category [7,9]. Reviews of physician and organizational barriers have similarly provided limited differentiation among nephrologists, vascular surgeons, and dialysis nurses [10]. Administrative cohort studies have identified predictors of access creation and use but cannot show how providers understand the clinical and service circumstances surrounding these patterns [11]. Qualitative studies have provided important accounts of patient experiences, whereas role-differentiated provider perspectives have received less attention [8].

Nephrologists, vascular surgeons, and dialysis nurses engage with patients at different stages of chronic kidney disease (CKD) care, counseling, referral, evaluation, access creation, and preparation for hemodialysis. Their professional responsibilities may therefore influence which barriers they prioritize and how they understand potential supports. Comparing these perspectives may identify both shared priorities and differences relevant to multidisciplinary service planning.

### Study rationale and objectives

A mixed-methods approach was used because structured ratings could describe the relative emphasis placed on barriers and supportive factors across professional groups, whereas open-ended responses could provide context for how providers understood these issues. The quantitative objective was to compare nephrologists’, vascular surgeons’, and dialysis nurses’ reported perceptions of patient-, provider-, and system-level barriers and supportive factors related to timely predialysis AV access preparation in Upper Northern Thailand. The qualitative objective was to describe the contextual considerations providers associated with selected barriers, role-specific obstacles, and provider-suggested actions. Through integration, the study aimed to identify areas of shared priority and to clarify how the meanings attached to those priorities varied across professional roles.

## Methods

### Study design

This cross-sectional study used a convergent mixed-methods design to examine and compare nephrologists’, vascular surgeons’, and dialysis nurses’ perceptions of barriers and supportive factors related to timely predialysis AV access preparation in Upper Northern Thailand. Quantitative and qualitative data were collected concurrently through a single self-administered survey containing Likert-scale items and open-ended questions. The two datasets were analyzed separately and integrated during interpretation [12,13]. The quantitative component was used to describe and compare response patterns across professional groups, while the qualitative component provided context for selected barriers and role-specific obstacles and elicited provider-suggested actions.

In this study, *“*predialysis AV access preparation*”* is used as an umbrella term encompassing planning and, when clinically appropriate, surgical creation of an AVF or AVG before hemodialysis initiation, consistent with individualized ESKD Life-Plan framework.

### Questionnaire development

#### Item generation.

Initial exploratory qualitative interviews were conducted with a convenience sample of healthcare providers representing the target population, including five dialysis nurses, five vascular surgeons, and two nephrologists. These interviews explored perceived barriers to timely predialysis AV access planning and creation, as well as suggestions for improvement. Responses were documented in written form, de-identified, and synthesized into conceptual categories. Conceptual categories were then mapped to candidate questionnaire items by the research team, with items retained when they reflected recurring provider-identified barriers or potential supports and could be rated across one or more professional groups. Based on these findings, the research team developed a structured survey instrument comprising ten core barrier items spanning patient-, provider/team-, and system-level domains; six supportive-factor items; profession-specific obstacle items tailored to nurses, surgeons, and nephrologists; and three open-ended questions addressing factors that could support timely predialysis AV access preparation, urgent obstacles, and suggested improvements. This iterative, inductive process was designed to ensure content relevance and alignment with provider-reported clinical and service experiences.

### Content validation and pilot testing

The draft questionnaire was reviewed by three subject-matter experts in clinical nephrology, vascular access surgery, and mixed-methods research to assess content validity, clarity, domain coverage, and relevance across the three professional groups. Revisions were made to improve item wording, reduce ambiguity, and ensure that role-specific items were appropriate for dialysis nurses, vascular surgeons, and nephrologists. Pilot testing was then conducted with six providers to evaluate feasibility, comprehension, completion time, and the clarity of instructions and response options. Feedback was used to refine item wording and survey format before final administration. The questionnaire was developed for descriptive use in this exploratory provider-perception study and was not intended as a fully validated psychometric instrument.

### Participants, setting, and recruitment

Eligible participants were healthcare providers directly involved in CKD management, vascular access planning and coordination, KRT education, and hemodialysis care, including dialysis nurses, vascular surgeons, and nephrologists. In some regional hospital settings, dialysis nurses participate in predialysis KRT counseling, including education for patients and families regarding dialysis modalities, advantages and disadvantages of peritoneal dialysis and hemodialysis, and preparation for the transition to dialysis initiation. The study was conducted in Upper Northern Thailand (Health Region 1), which comprises eight provinces: Chiang Mai, Chiang Rai, Lamphun, Lampang, Phrae, Nan, Phayao, and Mae Hong Son. Recruitment took place between February and April 2025 using a pragmatic, opportunity-based approach. Participants were recruited primarily through a regional academic meeting on vascular access held at the Faculty of Medicine, Chiang Mai University, supplemented by targeted invitations distributed through established professional networks to nephrologists, vascular surgeons, and dialysis nurses practicing in the region. Participation was voluntary and anonymous, and no financial or material incentives were provided.

A formal sample size calculation was not undertaken because the study was exploratory and was not designed to test a predetermined effect size. The final sample comprised 78 dialysis nurses, 19 vascular surgeons, and 16 nephrologists and was used for descriptive cross‑professional comparisons and identification of recurring concepts in the open-ended responses. The same participants contributed to both the closed-ended and open-ended components of the survey.

### Ethical approval

This study was conducted as part of the research project *“The Registry for Vascular Access in Patients Requiring Hemodialysis in the Northern Thai Population”*. Ethical approval was obtained from the Research Ethics Committee of the Research Institute for Health Sciences, Chiang Mai University (Approval No. HEC 79/2024; 17 December 2024). Written informed consent was waived because data were collected anonymously, with consent implied by voluntary questionnaire completion.

### Data collection procedures

The final questionnaire consisted of five sections: provider characteristics; core barrier items (10 Likert-scale items); profession-specific obstacle items (14 Likert-scale items); supportive factor items (6 Likert-scale items); and open-ended questions. Participants completed the questionnaire independently at a single time point. Anonymous participation and the absence of incentives were intended to encourage candid responses. The qualitative component comprised brief written responses to three open-ended survey questions rather than in-depth interviews or focus groups. Overall, 97 of 113 participants provided at least one open-ended response; 94 responded to the question on factors that could support timely predialysis AV access preparation, 92 identified an urgent obstacle, and 68 suggested actions for improvement.

### Quantitative measures and analysis

Categorical variables were summarized using frequencies and percentages, and Likert-scale items were summarized using medians and interquartile ranges (IQRs). Core barrier and supportive-factor responses were compared across professional groups using Kruskal–Wallis tests. Because the questionnaire was newly developed and the Likert responses were ordinal, the primary quantitative interpretation was conducted at the item level.

Participant-level domain summaries were calculated by averaging responses to conceptually related items within each predefined domain; medians and IQRs of these summaries were reported descriptively. Domain summaries were not used to select items for the joint displays or to derive the principal integrated findings. There were no missing responses for core barrier, profession-specific obstacle, or supportive-factor items; non-response occurred only for some open-ended questions. Exact Kruskal–Wallis p-values are reported in S3 File. Given the exploratory analyses, multiple item comparisons, and small professional subgroups, p-values were not adjusted for multiplicity and were interpreted descriptively rather than as confirmatory evidence of between-group differences.

Internal consistency was assessed using Cronbach’s alpha. For core barrier items, alpha was 0.67 for the patient-level domain (5 items), 0.71 for the system-level domain (4 items), and 0.82 for the overall barrier scale (10 items). Given its modest internal consistency, the patient-level barrier domain was interpreted descriptively and was not treated as a validated unidimensional patient-readiness construct. For profession-specific obstacle items, Cronbach’s alpha was 0.84 for nurses (4 items), 0.77 for surgeons (5 items), and 0.89 for nephrologists (5 items). Supportive-factor domains demonstrated acceptable to good internal consistency: 0.79 for care coordination (2 items), 0.83 for system capacity and training resources (2 items), 0.77 for policy and financial support (2 items), and 0.87 for the overall supportive-factor scale (6 items). The questionnaire development process, content validation procedures, pilot-testing summary, and full questionnaire are provided in S1 File.

### Qualitative analysis

Open-ended responses were analyzed using a combined deductive–inductive approach. Given the brief written format, the analysis was intended to identify recurring concepts and provide contextual detail for the quantitative response patterns rather than to develop an in-depth interpretive theory. Initial codes were informed by the quantitative domains, while additional codes were developed from the responses. Multiple codes could be assigned when a response addressed more than one concept, but each respondent contributed no more than one instance of a code within a theme.

Clinical, technical, or setting-specific considerations that could not be meaningfully incorporated into the broader themes were retained as a separate context-specific theme. Coding decisions, theme definitions, and representative excerpts were reviewed iteratively by the research team. The complete coding framework and analytic process are provided in S2 File.

### Mixed-methods integration and selection of joint display items

Mixed-methods integration was conducted at the interpretation stage using a convergent approach, consistent with established procedures [12]. Quantitative and qualitative findings were brought together in joint displays (Tables 3–5), in which qualitative themes and excerpts were linked to selected quantitative items on the basis of conceptual relevance. This approach allowed the open-ended responses to provide contextual detail for the observed rating patterns.

Quantitative items were selected from the complete questionnaire dataset using predefined analytic criteria. Table 3 included barrier items with median ratings ≥4.0 in at least two professional groups and relevant qualitative material; comorbidity affecting clinical suitability was retained as a prespecified analytic exception because it was prominent in the qualitative responses. Table 4 included the highest-rated profession-specific obstacle for each provider group. Table 5 included supportive factors with median ratings ≥4.0 across all three groups and relevant provider-suggested actions.

The joint displays are illustrative rather than exhaustive and were not intended to rank barriers or supportive factors by prevalence or importance. Qualitative excerpts were used to show how participants described the selected items and were not treated as frequency estimates. Complete item-level results, p-values, and the rationale for item selection are provided in S3 File.

### Reflexivity and analytic rigor

A reflexivity and analytic rigor statement detailing researcher positioning, analytic decision-making, and strategies to enhance credibility and transparency is provided in S4 File.

## Results

### Participant characteristics

A total of 113 healthcare providers participated in the study, comprising 78 dialysis nurses, 19 vascular surgeons, and 16 nephrologists. Nurses reported the longest duration of professional experience (median 12 years, IQR 6–18), while surgeons reported the shortest (median 2 years, IQR 1–9). Practice settings varied by professional role. Nearly half of surgeons were based in university hospitals (47%), whereas none of the nurses and 13% of nephrologists practiced in this setting. Most nurses (62%) and nephrologists (75%) worked in government or provincial hospitals, with the remaining participants practicing in private hospitals or clinics. Clinical workload was summarized using role-specific measures collected in the questionnaire. Nurses reported a median of 120 dialysis patients under their care per month (IQR 64–275), surgeons reported a median of nine AV access procedures per month (IQR 5–12), and nephrologists managed a median of 150 patients with CKD stages 4–5 per month (IQR 58–200). Maintenance-hemodialysis workload was not collected for nephrologists. The median provider-estimated proportion of patients receiving timely predialysis AV access planning and creation was 20% for each professional group. These estimates describe respondents’ practice contexts and should not be interpreted as audited institutional rates. Detailed participant characteristics are presented in Table 1.

**Table 1:** Characteristics of participating healthcare providers.

Characteristic	Nurses (n = 78)	Surgeons (n = 19)	Nephrologists (n = 16)
Years in role, median (IQR)	12 (6–18)	2 (1–9)	5 (2–14.5)
Workplace — University hospital, n (%)	0 (0%)	9 (47%)	2 (13%)
— Government/provincial hospital, n (%)	48 (62%)	9 (47%)	12 (75%)
— Private sector (hospital or clinic), n (%)	30 (38%)	1 (6%)	2 (12%)
Role-specific workload measure			
—Dialysis patients under care/month — median (IQR)	120 (64–275)	—	—
—AV access procedures/month — median (IQR)	—	9 (5–12)	—
—CKD 4–5 patients under care/month — median (IQR)	—	—	150 (58–200)
Estimated % of patients receiving timely predialysis AV access planning and creation— median (IQR)	20 (7–73)	20 (10–50)	20 (10–28)

Note: IQR = interquartile range. Values are presented as median (IQR) or n (%) as indicated. Dashes (–) indicate non-applicable roles. Workload measures were role-specific. Nephrologists’ active maintenance hemodialysis patient workload was not collected. These provider-estimated proportions of predialysis AV access were self-reported by providers and are presented as contextual practice estimates, not audited institutional rates.

### Qualitative themes framing barriers and supportive considerations

Analysis of open-ended responses identified five themes: Patient and Family Readiness, Care Pathway Coordination, System Capacity and Access, Policy and Financial Support, and Context-specific Technical or Clinical Suitability Considerations. These themes organized the contextual material used in the subsequent joint displays. Definitions and representative quotations are presented in Table 2, with the complete coding framework provided in S2 File.

**Table 2:** Qualitative themes framing barriers and supportive considerations across professional roles, identified from open-ended responses.

Theme	Definition	Representative Quote (ID, role)
**Patient & Family Readiness**	Knowledge, readiness, and beliefs influencing acceptance of timely predialysis AV access preparation	“Adequate knowledge helps patients recognize the importance of AVF preparation before dialysis initiation.” (ID8, Nurse)
**Care Pathway Coordination**	Referral timing, interdisciplinary communication, and continuity across CKD progression and AV access preparation pathway	“Coordination between nephrologists and surgeons is often fragmented.” (ID12, Nurse)
**System Capacity & Access**	Resources, procedural wait time, staffing, and service availability	“Prolonged surgical waiting times discourage patients from proceeding with AVF creation.” (ID109, Nephrologist)
**Policy & Financial Support**	Coverage, reimbursement, and policy-level enabling conditions affecting access planning and service delivery	“Financial coverage and reimbursement policies play a critical role in enabling timely AVF creation.” (ID80, Surgeon)
**Context-specific** **Technical/Clinical Suitability Considerations**	Setting-dependent technical or clinical constraints influencing access selection and feasibility not captured by higher-level system or policy themes	“There is a shortage of specialized personnel, along with limited access to appropriate tools for vascular assessment.” (ID13, Nurse)

Note: ID = participant identifier. Illustrative quotations show how participants expressed each theme and are not frequency estimates. The complete coding framework and analytic details are provided in S2 File.

### Integrated barriers to timely predialysis AV access preparation

Table 3 integrates five selected barrier items with related qualitative material. These findings represent providers’ perceptions rather than direct patient-reported or audited clinical and service indicators. Complete quantitative results are provided in S3 File, part A.

**Table 3:** Joint display of selected provider-perceived barriers to timely predialysis arteriovenous access preparation.

Quantitative Barrier Item	Quantitative Domain	Quantitative Summary (Median Score N/S/Neph, IQR Overall)	Illustrative Quote (ID, role)	Linked Qualitative Theme
Patient understanding/ acceptance of timely predialysis AV access planning	Patient‑ level	Median 4.0 / 3.0 / 4.0 (IQR 3.0–5.0)	“Most patients lack full understanding and feel fear…” (ID4, Nurse)	Patient & Family Readiness
Late presentation or advanced CKD stage at first consultation	Patient‑ level	Median 4.0 / 4.0 / 4.0 (IQR 3.0–5.0)	“Patients often arrive late… leaving no time for AVF planning.” (ID108, Nephrologist)	Patient & Family Readiness
Procedural wait time for AV access	System‑ level	Median 4.0 / 3.0 / 4.0 (IQR 3.0–5.0)	“The wait for surgery is so long that patients just give up…” (ID30, Nurse)	System Capacity & Access
Comorbidities affecting clinical suitability for AV access	Patient‑ level	Median 4.0 / 3.0 / 2.5 (IQR 3.0–5.0)	“Some patients are not suitable due to health issues…” (ID6, Nurse)	Context-specific Technical/ Clinical Suitability Considerations
Insurance and reimbursement process affects AV access procedures	System‑ level	Median 4.0 / 4.0 / 3.5 (IQR 2.0–4.0)	“Some procedures are not reimbursed…” (ID80, Surgeon)	Policy & Financial Support

Note: N = nurse; S = surgeon; Neph = nephrologist. Quantitative summaries are presented as median scores (interquartile range). Barrier items were rated on a 5-point Likert scale (1 = not a barrier to 5 = very major barrier). Quantitative domains reflect the a priori structure of the questionnaire (patient-, provider/team-, and system-level). Qualitative excerpts are illustrative and provided for explanatory purposes; they are not intended to indicate prevalence, dominance, or relative importance.

Patient understanding and acceptance received median ratings of 4.0 among nurses and nephrologists and 3.0among surgeons. Open-ended responses associated this item with fear, misperceptions, and difficulty making decisions about AV access preparation. Late presentation received a median rating of 4.0 in all three groups; participants described limited time for counseling, evaluation, and access preparation when patients entered care with advanced CKD.

Procedural waiting time received median ratings of 4.0 among nurses and nephrologists and 3.0 among surgeons. Participants described delays in scheduling and difficulties maintaining patient engagement during prolonged waits. Insurance coverage and reimbursement processes received median ratings of 4.0 among nurses and surgeons and 3.5 among nephrologists, with qualitative responses referring to coverage limitations and administrative delays. Ratings for comorbidities affecting clinical suitability varied across groups, with medians of 4.0 among nurses, 3.0 among surgeons, and 2.5 among nephrologists. Qualitative responses described vascular suitability, comorbidities, and procedural complexity as relevant clinical considerations. These integrated quantitative summaries and illustrative qualitative excerpts are presented in Table 3.

### Selected role-specific obstacles

Table 4 presents the highest-rated profession-specific obstacle within each provider group together with related qualitative material. Full quantitative results for all role-specific obstacle items are provided in S3 File, part B. Among nurses, the ability to support patient and family self-care and readiness had a median rating of 3.0. Open-ended responses described uncertainty in modality decisions and the need for patient and family preparation.

**Table 4:** Joint display of selected role-specific obstacles.

Provider Role	Quantitative Obstacle Score(Median: IQR)	Top Role-Specific Obstacle Item	Illustrative Qualitative Quote (ID)	Linked Qualitative Theme
Nurse	3.0 (IQR 3.0–4.0)	Ability to support patient and family self-care readiness	“Difficulty for patients and families to decide on a dialysis modality often delays the entire AVF preparation process.” (ID34).	Patient & Family Readiness
Surgeon	3.0 (IQR 2.0–4.0)	Technical complexity of AV access procedures in high-risk or complex cases	“AVF creation can be technically challenging in patients with severe comorbidities or unsuitable vessels, such as those with prior venipuncture in the targeted arm.” (ID93)	Context-specific Technical/ Clinical Suitability Considerations
Nephrologist	3.5 (IQR 2.0–4.0)	Preparation of patients prior to AV access planning and creation (medical and psychological)	“Without early counseling and medical optimization, it is difficult to prepare patients physically and mentally for timely AVF creation.” (ID102)	Care Pathway Coordination

Note: Quantitative summaries are derived from structured role-specific obstacle ratings and are presented as median (interquartile range). Items were rated on a 5-point Likert scale (1 = not a barrier to 5 = very major barrier). Qualitative excerpts are illustrative and provided for explanatory purposes; they are not intended to indicate prevalence, dominance, or relative importance.

Among surgeons, technical complexity of AV access procedures in high-risk or complex cases had a median rating of 3.0. Related responses referred to vascular suitability, comorbidity, and anticipated procedural difficulty. Among nephrologists, preparation of patients prior to AV access planning or creation had a median rating of 3.5. Qualitative responses referred to counseling, medical preparation, and referral timing during CKD follow-up.

### Supportive factors and provider-suggested actions

Four supportive factors received median ratings of at least 4.0 across all three professional groups: predictable financial coverage and reimbursement mechanisms for AV access procedures, the presence of a dedicated vascular access coordinator, electronic referral and longitudinal follow-up systems, and proactive, stage-aligned AV access planning guidelines within the ESKD Life-Plan framework. Financial coverage and stage-aligned planning guidelines received the highest median ratings among nurses and nephrologists, while all four factors were rated positively across groups.

Open-ended responses linked these factors to patient preparation, referral and follow-up, access to procedures, and clarity of planning. The qualitative findings provided contextual detail on how providers understood these supportive factors. Table 5 presents illustrative provider-suggested actions corresponding to each factor. These actions were reported by participants and were not evaluated as interventions. Complete quantitative results for all supportive-factor items are provided in S3 File, part C.

**Table 5:** Joint display of supportive factors and provider-suggested actions.

Quantitative Supportive Factor	Quantitative Domain	Quantitative Summary (Median Score N/S/Neph, IQR Overall)	Illustrative Quote (ID, role)	Suggested Action (ID, role)	Linked Qualitative Theme
Presence of a dedicated vascular access coordinator	Care coordination	4.0 / 4.0 / 4.0 (IQR 3.0–5.0)	“A coordinator would help streamline referrals and follow-up.” (ID85, Surgeon)	“Assign a dedicated coordinator to track AV access (AVF and AVG) planning across CKD stages.” (ID27, Nurse)	Care Pathway Coordination
Electronic referral and longitudinal follow‑ up systems	System capacity and training resources	4.0 / 4.0 / 4.0 (IQR 3.0–5.0)	“An electronic system would make follow-up easier.” (ID4, Nurse)	“Implement e-referral to reduce delays.” (ID21, Nurse)	System Capacity & Access
Proactive, stage-aligned AV access planning guidelines within the ESKD Life-Plan framework	Policy and financial support	5.0 / 4.0 / 4.5 (IQR 4.0–5.0)	“Clear AV access guidelines help unify practice.” (ID92, Surgeon)	“Establish a proactive AVF and AVG planning policy across CKD stages.” (ID101, Nephrologist)	Policy & Financial Support
Predictable financial coverage and reimbursement mechanisms for AV access procedures	Policy and financial support	5.0 / 4.0 / 4.5 (IQR 3.0–5.0)	“Financial support and benefits are essential.” (ID23, Nurse)	“Strengthen reimbursement processes and clarify coverage criteria for AV access procedures.” (ID109, Nephrologist)	Policy & Financial Support

Note: N = nurse; S = surgeon; Neph = nephrologist. Quantitative summaries are presented as median scores (interquartile range). Supportive factor items were rated on a 5-point Likert scale (1 = no role to 5 = very important role). Qualitative excerpts and provider-suggested actions are illustrative and provided for explanatory purposes; they are not evaluated as interventions and are not intended to indicate prevalence, dominance, or relative importance.

## Discussion

This exploratory regional mixed-methods study integrated quantitative ratings and brief open-ended responses from dialysis nurses, vascular surgeons, and nephrologists in Upper Northern Thailand. Providers commonly identified limited patient understanding, late presentation, procedural waiting times, reimbursement constraints, and clinical suitability concerns as barriers to timely predialysis AV access preparation. They also endorsed predictable financial coverage and reimbursement mechanisms, a dedicated vascular access coordinator, electronic referral and longitudinal follow-up systems, and proactive, stage-aligned AV access planning guidelines as supportive strategies. The qualitative responses added context to these rating patterns and showed that, although providers shared several priorities, they emphasized different role-related considerations. These findings represent provider-reported, hypothesis-generating insights intended to inform future service evaluation.

### Shared and varying perceptions of patient, system, and clinical barriers

Providers commonly identified limited patient understanding and late presentation as barriers to timely predialysis AV access preparation. Patient understanding and acceptance received relatively high ratings from nurses and nephrologists, while late presentation was rated highly across all three professional groups. In the open-ended responses, providers linked limited understanding to fear, uncertainty, and incomplete awareness of CKD progression. Late presentation was described as reducing the time available for counseling, clinical evaluation, and access preparation. These accounts are consistent with qualitative studies reporting that fear of cannulation or complications and limited understanding of CKD progression may contribute to delayed or deferred AV access planning [7,14]. Previous studies have also associated late nephrology referral and the absence of structured predialysis pathways with hemodialysis initiation before permanent access is available [11,15].

Procedural waiting times and insurance or reimbursement constraints were also perceived as important system-level barriers. Quantitative ratings indicated concern across professional groups, while the open-ended responses referred to scheduling delays, limited procedural capacity, and uncertainty regarding coverage or administrative approval. These accounts are consistent with previous reports of operating-room limitations, referral difficulties, and administrative barriers to permanent AV access placement [9,16]. Because patients and families were not directly surveyed, the patient-level findings represent providers’ interpretations of patient readiness and decision-making rather than direct patient-reported evidence.

Perceptions of clinical suitability varied more markedly across professional groups. Nurses rated comorbidity-related barriers more highly than surgeons and nephrologists, while the qualitative responses referred to vascular anatomy, comorbidity burden, and clinical stability as considerations relevant to AV access creation. Previous studies have reported associations between AV access failure or nonmaturation and cardiovascular disease, peripheral artery disease, diabetes, and body mass index [17–19]. The present study did not directly examine why these ratings differed across professional groups. These patterns may reflect differences in professional responsibilities, clinical exposure, or the stages at which providers encounter patients and warrant further investigation using objective clinical and service data.

Together, these findings indicate that providers perceived barriers at patient, system, and clinical levels, with both shared concerns and variation in professional emphasis. They identify areas for prospective evaluation using patient-reported information, referral and scheduling records, reimbursement data, and AV access outcomes.

### Role-related variation in perceived obstacles

Within each professional group, the selected profession-specific items and accompanying written responses highlighted different aspects of AV access preparation. These are descriptive because the items differed across professions, subgroup sizes were uneven, and the three sets of role-specific items were not directly compared statistically.

For dialysis nurses, the ability to support patient and family self-care and readiness was selected as the highest-rated role-specific obstacle. Written responses referred to uncertainty about dialysis modality decisions, the importance of family involvement, and difficulties preparing patients before dialysis became urgent. In some hospitals in Upper Northern Thailand, dialysis nurses also participate in predialysis KRT counseling, although the extent of this involvement was not measured for individual respondents. The nursing findings may therefore reflect varying combinations of experience with predialysis counseling and with patients transitioning to hemodialysis, rather than the full range of nursing roles across the predialysis pathway. Previous research has similarly emphasized the importance of patient and family engagement in permanent access planning [7].

For vascular surgeons, the selected obstacle concerned the technical complexity of AV access procedures in high-risk or complex cases. Written responses referred to vascular suitability, comorbidity burden, and anticipated procedural difficulty. These concerns are consistent with evidence that vascular anatomy and clinical risk influence access selection, maturation, and usability [20]. In the present study, technical and clinical feasibility formed a prominent part of surgeons’ accounts, although the frequency with which these considerations prevented access creation was not assessed.

For nephrologists, the selected obstacle concerned patient preparation prior to AV access planning and creation. Written responses referred to medical optimization, counseling, referral timing, and uncertainty in anticipating dialysis initiation. Previous studies have likewise described challenges related to referral thresholds and the timing of access preparation during CKD progression [21]. The present study did not include objective measures of referral decisions, CKD trajectories, or referral-to-procedure intervals.

Taken together, these within-role findings suggest that providers emphasized different aspects of AV access preparation in relation to their professional responsibilities and points of contact with patients. The patterns do not necessarily indicate disagreement across professions; rather, they show how shared priorities may be understood through different professional lenses. This interpretation generates testable questions for future studies of communication, referral processes, task allocation, and clinical outcomes across the AV access pathway.

### Shared endorsement of supportive strategies and priorities for evaluation

Providers across all three professional groups consistently endorsed four supportive strategies: predictable financial coverage and reimbursement mechanisms, a dedicated vascular access coordinator, electronic referral and longitudinal follow-up systems, and proactive, stage-aligned AV access planning guidelines. Open-ended responses linked these strategies to patient preparation, referral and procedural coordination, and continuity of follow-up. Together, these findings indicate shared provider support and identify priorities for future service evaluation.

Predictable financial coverage and reimbursement mechanisms were among the highest-rated supportive factors across professional groups. Written responses linked financial support to greater certainty regarding procedure coverage and patients’ ability to proceed with access preparation. Previous studies have likewise identified financial and administrative barriers as relevant to permanent access placement [16]. In the present study, financial coverage was identified as a provider priority, although reimbursement pathways, approval times, out-of-pocket costs, and their relationships with AV access outcomes were not measured.

Providers also endorsed the presence of a dedicated vascular access coordinator and electronic referral and longitudinal follow-up systems. Qualitative responses associated these strategies with patient tracking, communication between services, referral follow-up, and monitoring during CKD progression. These proposed functions are consistent with the broader literature on coordinated vascular access care [22]. Future studies should define the coordinator’s responsibilities, examine how electronic systems could be incorporated into local services, and assess their effects on referral and access outcomes.

Proactive, stage-aligned AV access planning guidelines also received high ratings across professional groups. Participants associated clearer criteria and earlier planning with counseling and referral decisions. This interpretation is consistent with the individualized ESKD Life-Plan approach, in which access decisions are reviewed according to clinical trajectory and patient circumstances rather than through a uniform AVF-first rule [1]. The findings therefore support the evaluation of locally adapted planning guidelines, including appropriate referral triggers and their effects on timely AV access preparation.

Overall, convergence in provider ratings identified a shared set of strategies for service development in Upper Northern Thailand. The qualitative findings added context by showing the functions providers associated with these strategies, without indicating that the strategies were already in place or operated differently across professions. Future evaluations should assess acceptability, feasibility, role allocation, and effects on referral-to-procedure time, AV access creation before dialysis, and access status at hemodialysis initiation. Fig 1 summarizes these provider-perceived relationships as an illustrative, hypothesis-generating framework.

**Fig 1:**
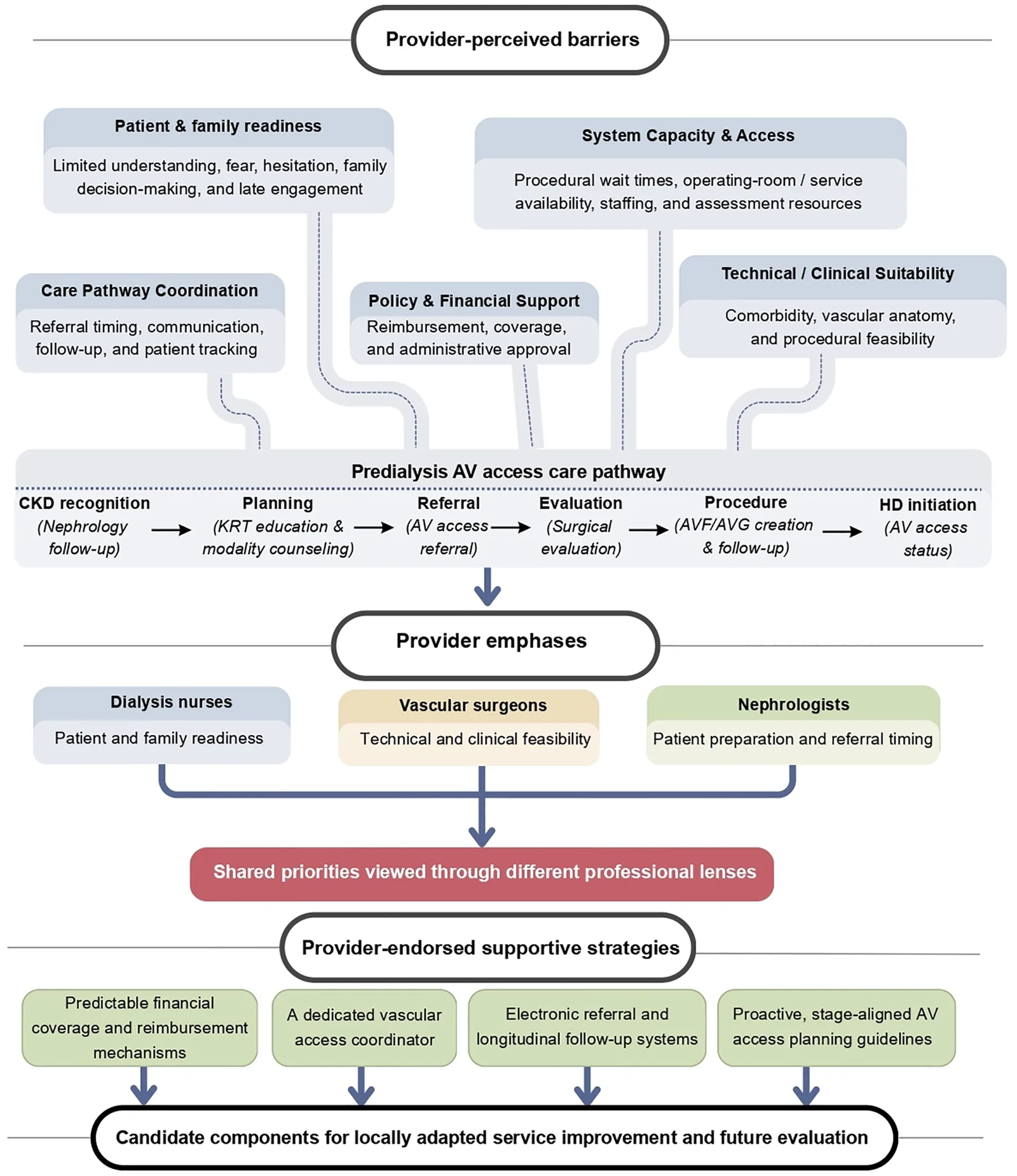
Conceptual summary of provider-perceived barriers, role-related emphases, and supportive strategies in predialysis AV access preparation. The figure integrates provider-reported quantitative and qualitative findings across the predialysis AV access care pathway. Providers shared several priorities but emphasized different aspects according to professional role. The provider-endorsed strategies shown are candidate components for locally adapted service improvement and future evaluation. The figure is illustrative and does not represent a causal pathway or observed service implementation.

### Implications for service improvement and future evaluation

The findings identify several provider-perceived priorities that may inform locally adapted service improvement in Upper Northern Thailand. At the clinical level, providers’ emphasis on patient understanding, readiness, medical preparation, and clinical suitability suggests that structured counseling and anticipatory preparation during advanced CKD care warrant further evaluation. These approaches should remain individualized and consistent with the ESKD Life-Plan framework, rather than assume that AVF creation is appropriate for every patient [1].

At the organizational and system levels, predictable financial coverage and reimbursement mechanisms, the presence of a dedicated vascular access coordinator, electronic referral and longitudinal follow-up systems, and proactive, stage-aligned AV access planning guidelines represent candidate components for locally adapted service improvement. Initial evaluation should examine their acceptability, feasibility, role definition, and fit with existing referral, procedural, and reimbursement arrangements. Subsequent prospective studies could assess their effects on measurable outcomes, including completion of counseling and referral, referral-to-procedure time, AV access creation before dialysis, and access status at hemodialysis initiation.

The findings also suggest that future service initiatives should involve dialysis nurses, nephrologists, vascular surgeons, patients, and families in their design. Co-design may help align counseling, coordination, and referral components with local professional responsibilities and patient needs, while allowing the different perspectives identified in this study to inform intervention design. These context-specific implications provide priorities for local development and evaluation rather than prescriptive national policy recommendations.

### Strengths and limitations

This study has several strengths. The convergent mixed-methods design enabled integration of item-level quantitative patterns with brief written responses that added contextual detail to providers’ ratings. Including dialysis nurses, vascular surgeons, and nephrologists captured perspectives from three professional groups involved at different stages of AV access care. The use of predefined criteria for the illustrative joint displays, together with the complete item-level quantitative results in S3 File and the qualitative coding framework in S2 File, strengthened the transparency of the integrated analysis.

Several limitations should also be considered. First, the study examined provider-reported perceptions rather than direct patient or family experiences, observed care processes, or objective clinical outcomes. Self-reported responses may have been affected by recall, reporting, or social desirability bias. Patient-level items should therefore not be interpreted as patient-reported determinants of readiness or decision-making. In addition, the modest internal consistency of the patient-level domain supports treating these items as heterogeneous provider-perceived concerns rather than as a validated patient-readiness construct. The qualitative component consisted of brief written responses rather than in-depth interviews or focus groups and therefore provided contextual breadth but limited interpretive depth.

Second, the professional subgroups were uneven, with dialysis nurses comprising most participants and relatively small numbers of nephrologists and vascular surgeons. Profession-specific items were administered only to the corresponding provider group and were not directly compared across professions; apparent role-related differences should therefore be interpreted descriptively. In some regional hospitals, dialysis nurses participate in predialysis counseling, whereas in other settings their perspectives may arise primarily from caring for patients transitioning to hemodialysis. The extent of individual nurses’ involvement in predialysis care was not measured, and dedicated CKD or predialysis nurses were not sampled separately. The nursing findings therefore should not be considered representative of all nursing roles in the predialysis pathway.

Third, pragmatic recruitment through a regional vascular access meeting and professional networks may have introduced selection bias by preferentially including providers with greater interest or engagement in vascular access care. Participants were drawn from Upper Northern Thailand, where service organization, workforce capacity, referral arrangements, and reimbursement processes may differ from those in other regions. The findings are therefore context-specific and are not representative of all providers in Thailand.

Finally, the study did not include objective measures of catheter use at dialysis initiation, audited AVF or AVG creation rates, referral completion, referral-to-procedure intervals, failed referrals, procedural waiting time, reimbursement delays, or patient-level reasons for noncompletion. Provider-estimated predialysis access percentages were contextual estimates rather than audited institutional rates. The study also did not identify a clinically eligible “delayed-but-eligible” patient cohort or evaluate the feasibility, implementation, or effectiveness of the endorsed strategies. Accordingly, the findings identify provider-perceived barriers and priorities for future evaluation rather than determinants of actual delayed access creation. Future studies should integrate provider and patient perspectives with clinical records, referral and scheduling data, reimbursement information, and access status at hemodialysis initiation.

## Conclusion

In this exploratory regional provider-perception study, healthcare providers in Upper Northern Thailand identified patient-, clinical-, care pathway-, system-, and policy-level barriers to timely predialysis AV access preparation. Across professional groups, providers shared several priority barriers and supportive strategies while emphasizing different role-related considerations. These context-specific, hypothesis-generating findings may inform locally adapted service improvement and the design of future evaluations. Further studies integrating provider and patient perspectives with clinical and care-pathway data are needed to assess the feasibility and effects of the proposed strategies on timely AV access preparation and access status at hemodialysis initiation.

### What this paper adds

This paper provides exploratory regional evidence on provider perspectives regarding timely predialysis arteriovenous access preparation by comparing nephrologists, vascular surgeons, and dialysis nurses in Upper Northern Thailand. Providers identified barriers related to patient and family readiness, care pathway coordination, system capacity and access, policy and financial support, and clinical suitability. Although professional groups shared several barriers and supportive factors, the qualitative findings showed that they attached different role-related considerations to these priorities. The study also highlights four provider-endorsed strategies for future evaluation: predictable financial coverage and reimbursement mechanisms, a dedicated vascular access coordinator, electronic referral and longitudinal follow-up systems, and proactive, stage-aligned AV access planning guidelines. These context-specific findings may inform locally adapted service improvement and future evaluation.

## Supporting information

S1 File Questionnaire development, content validation, pilot testing, and full questionnaire. (PDF)

S2 File Qualitative Coding Framework for Open-Ended Responses. (PDF)

S3 File Full List of Quantitative Items and Descriptive Statistics. (PDF)

S4 File Reflexivity and Analytic Rigor Statement. (PDF)
