# Supplementary material for: Multidisciplinary providers’ perspectives on timely predialysis arteriovenous access preparation: An exploratory mixed-methods study in upper Northern Thailand
Source: PLoS One. 2026 Oct 5;21(10):e0359863. doi: 10.1371/journal.pone.0359863 (PMC13637856; doi:10.1371/journal.pone.0359863)
Supplement: Supplementary file 1 [file pone.0359863.s001.pdf]

Supporting Information

S1 File. Questionnaire development, content validation, pilot testing, and full questionnaire

Questionnaire development and content validation process

Initial exploratory qualitative elicitation

The questionnaire was developed through an iterative process beginning with an initial exploratory qualitative elicitation phase. A semi-structured guide was prepared for the three target professional groups involved in the predialysis AV access pathway: dialysis nurses, vascular surgeons, and nephrologists. This initial phase combined interviewer-facilitated discussion with anonymous written responses in the guide document; respondents were not required to provide their names.

For dialysis nurses, the guide explored professional role, workplace, years of experience related to AVF care, involvement in patient education regarding self-care and dialysis modality options, content and format of education provided, assessment of patient understanding after education, perceived barriers to preemptive AVF creation before catheter use, and suggestions to support patients in receiving AVF before hemodialysis initiation.

For vascular surgeons, the guide explored professional role, workplace, years of experience related to AVF care, usual CKD stage at referral, waiting time for surgery, perceived barriers to preemptive AVF creation, and suggestions to support AVF creation before first hemodialysis.

For nephrologists, the guide explored professional role, workplace, years of experience related to AVF care, perceived barriers to preemptive AVF creation, and suggestions to support AVF creation before first hemodialysis.

Responses from this initial exploratory phase were documented in written form, de-identified, and synthesized into conceptual categories reflecting patient-level, provider/team-level, system-level, policy/financial, and clinical or technical suitability considerations.

Translation of exploratory findings into questionnaire items

The research team mapped the conceptual categories derived from the initial exploratory phase to candidate questionnaire items. Items were retained when they reflected recurring provider-identified barriers or potential supports, were relevant to timely predialysis AV access planning and creation, and could be rated across one or more professional groups.

This process resulted in a structured self-administered questionnaire comprising:

1. provider characteristics;
2. ten core barrier items spanning patient-level, provider/team-level, and system-level domains;
3. fourteen profession-specific obstacle items tailored to dialysis nurses, vascular surgeons, and nephrologists;

4. six supportive-factor items addressing care coordination, system capacity/training resources, and policy/financial support; and
5. three open-ended questions addressing "top facilitators," the most urgent obstacle, and suggested improvements.

Although the initial exploratory guide used the term "preemptive AVF," the final questionnaire used the broader wording "timely predialysis AV access planning and creation" to encompass both AVF and AVG where clinically appropriate. In the manuscript, these processes are collectively referred to as "predialysis AV access preparation."

Content validation and pilot testing

The draft questionnaire was reviewed by three subject-matter experts in clinical nephrology, vascular access surgery, and mixed-methods research. The expert review assessed content validity, clarity, domain coverage, and relevance across the three professional groups. Revisions were made to improve wording, reduce ambiguity, and ensure that role-specific items were appropriate for dialysis nurses, vascular surgeons, and nephrologists.

Pilot testing was then conducted with 6 providers (2 dialysis nurses, 2 vascular surgeons, and 2 nephrologists) to evaluate feasibility, comprehension, completion time, clarity of instructions, and clarity of response options. Feedback from pilot testing was used to refine item wording and format before final administration.

The questionnaire was developed for descriptive use in this exploratory provider-perception study. Cronbach's alpha was used to assess internal consistency of predefined item domains; however, internal consistency alone was not considered evidence of full psychometric validation.

Provider Questionnaire on Barriers and Facilitators to Predialysis Arteriovenous (AV) Access Planning and Creation

Questionnaire overview

This study used a self-administered questionnaire to assess health care providers' perceptions of barriers and facilitators to timely predialysis arteriovenous access (AV access) planning and creation prior to hemodialysis initiation. The questionnaire was administered to three provider groups involved in the vascular access pathway: dialysis nurses, vascular surgeons, and nephrologists.

The instrument incorporated **quantitative Likert-type items and qualitative open-ended** questions and was used within the convergent mixed-methods design.

Section 1. Provider characteristics

The following information was collected from all respondents:

1. **Professional role**
   - □ Dialysis nurse
   - □ Vascular surgeon
   - □ Nephrologist
2. **Years of experience in current position** (years)
3. **Primary workplace**
   - □ University hospital
   - □ Government/provincial hospital
   - □ Private hospital
   - □ Private clinic
   - □ Other (specified by respondent)
4. **Role-specific monthly clinical workload**
   - □ Nurses: number of dialysis patients under care per month_____
   - □ Surgeons: number of AV access procedures (AVF or AVG) performed per month_____
   - □ Nephrologists: number of patients with CKD stage 4–5 under care per month_____
5. Estimated proportion (%) of patients receiving timely predialysis AV access planning and creation in the respondent's practice_____

Section 2. Core barriers to timely predialysis AV access planning and creation

All respondents rated 10 core barrier items covering patient-, provider-, and system-level domains.

Each item was rated using a **5-point Likert-type scale**
(1 = not a barrier, 5 = very major barrier).

Patient-level barriers domain

1. Patient understanding and acceptance of timely predialysis AV access planning_____
2. Late presentation or advanced CKD stage at first consultation_____
3. Financial and travel constraints_____
4. Comorbidities affecting clinical suitability for AV access_____
5. Adherence and reliability of follow-up_____

Provider/team-level barriers domain

6. Coordination among multidisciplinary team members (nurses, nephrologists, surgeons)_____

System-level barriers domain

7. Procedural waiting time for AV access_____
8. Insurance coverage and reimbursement processes_____
9. Availability of surgical resources (operating room, personnel, equipment)_____
10. Efficiency of referral and follow-up systems_____

Note:

These items were later summarized and selectively integrated with qualitative data to construct joint displays highlighting convergent and divergent barriers across professional roles.

Section 3. Profession-specific role obstacles

Profession-specific obstacle items were tailored to the distinct clinical responsibilities of each provider group and were analyzed separately.

All items used the same **5-point Likert-type scale**
(1 = not a barrier, 5 = very major barrier).

Nurse-specific obstacles (4 items)

Patient-level barriers domain

1. Effectiveness of patient education and AV access preparation programs_____
2. Ability to support patient and family self-care and readiness_____

Provider-level barriers domain

3. Coordination within the multidisciplinary team during patient follow-up_____
4. Competency in assessing AV access maturation prior to cannulation_____

<u>Surgeon-specific obstacles (5 items)</u>

Provider-level barriers domain

1. Coordination with referring nephrologists_____
2. Systems for postoperative complication monitoring and management_____
3. Technical complexity of AV access procedures in high-risk or complex cases_____

System-level barriers domain

4. Operating theatre scheduling and resource management_____
5. Preoperative vascular assessment and vessel quality evaluation_____

<u>Nephrologist-specific obstacles (5 items)</u>

Provider-level barriers domain

1. Clarity of criteria for selecting candidates for timely predialysis AV access_____
2. Backup planning when AV access (e.g., AVF or AVG) creation is not feasible or unsuccessful_____
3. Determination of optimal timing for surgical referral_____
4. Preparation of patients prior to AV access planning and creation (medical and psychological)_____

System-level barriers domain

5. Burden of monitoring CKD progression and predicting dialysis initiation_____

Note:

Role-specific obstacle scores were analyzed separately within each professional group. Domain-level summaries were descriptive, and the highest-rated item for each group was selected for the joint display in Table 4.

<u>Section 4. Supportive factors for timely predialysis AV access planning and creation</u>

All respondents rated the perceived importance of six supportive factors.

Items were rated on a **5-point Likert-type scale**
(1 = no role, 5 = very important role).

Care coordination domain

1. Presence of a dedicated vascular access coordinator_____
2. Inter-institutional collaboration and referral networks_____

System capacity and training resource domain

3. Electronic referral and longitudinal follow-up systems_____
4. Ongoing training programs related to AV access care_____

Policy and financial support domain

5. Predictable financial coverage and reimbursement mechanisms for AV access procedures_____

6. Proactive, stage-aligned AV access planning guidelines within the ESKD Life-Plan framework_____

Note:

Supportive factors with consistently high ratings across roles were prioritized for integration with qualitative provider-suggested actions and operational implications.

Section 5. Open-ended questions

Respondents provided responses to the following open-ended questions:

1. **Top facilitators**

What are the three most important factors that could increase the rate of timely predialysis AV access planning and creation?

____

____

____

2. **Most urgent obstacle**

What is the most urgent obstacle that should be addressed to improve timely predialysis AV access planning and creation?

____

____

____

3. **Suggestions for improvement**

What specific suggestions do you have to improve the predialysis AV access care pathway in your setting?

____

____

____

Notes

- *Participation was voluntary and anonymous.*
- *The questionnaires was administered in Thai; this appendix provides the English-language description of questionnaire content.*
- *Quantitative item presented in Tables 3–5 of the main manuscript were selected using the predefined criteria described in Methods and S3 File. Complete item-level results and selection rationales are provided in S3 File.*
