# Supplementary material for: Multidisciplinary providers’ perspectives on timely predialysis arteriovenous access preparation: An exploratory mixed-methods study in upper Northern Thailand
Source: PLoS One. 2026 Oct 5;21(10):e0359863. doi: 10.1371/journal.pone.0359863 (PMC13637856; doi:10.1371/journal.pone.0359863)
Supplement: Supplementary file 2 [file pone.0359863.s002.pdf]

Supporting Information

S2 File. Qualitative coding framework for open-ended responses

This Supporting Information file documents the qualitative coding framework used to analyze open-ended responses from provider questionnaires and to derive the five qualitative themes presented in **Table 2**, supporting transparency of the qualitative component and its mixed-methods integration.

Data source and unit of analysis

Open-ended responses were collected from nurses, vascular surgeons, and nephrologists. The unit of analysis was a meaningful idea segment; individual responses could contain multiple segments reflecting distinct concepts. Multiple codes could be assigned when a response addressed more than one concept, but each respondent contributed no more than one instance of a code within a theme.

Coding and theme development

A combined deductive–inductive, codebook-based content analysis was conducted. Initial codes were informed by the quantitative questionnaire domains, while additional codes were developed inductively through close reading of the open-ended responses. Codes were then clustered into conceptually similar groups and organized into five higher-order themes: Patient and Family Readiness, Care Pathway Coordination, System Capacity and Access, Policy and Financial Support, and Context-specific Technical/Clinical Suitability Considerations.

Themes were refined through iterative review to preserve conceptual clarity and support explanatory integration with quantitative findings rather than frequency-based interpretation.

Cross-mapping and use of qualitative data

Codes were permitted to map to multiple themes when conceptually relevant, reflecting the interconnected nature of real-world care pathways. In the joint displays **(Tables 3–5)**, qualitative excerpts were used illustratively to contextualize quantitative patterns and were not intended to indicate prevalence, dominance, or relative importance.

Codebook of themes and codes used in Table 2

Theme name	Code	Brief definition<br>(analytic scope of the code)
**Theme 1. Patient and Family Readiness**	Patient knowledge and understanding of the need for timely predialysis AV access preparation	Statements emphasizing patient awareness or understanding of the need for timely predialysis AV

		access preparation before hemodialysis initiation.
	Patient readiness and acceptance of timely predialysis AV access preparation	Willingness, psychological readiness, acceptance of diagnosis or disease trajectory, and readiness to act, including overcoming fear or avoidance.
	Family awareness and support for timely predialysis AV access preparation	Family involvement, encouragement, decision support, and practical assistance that enables timely AV access planning.
	Insufficient patient education or awareness of timely predialysis AV access preparation	Gaps in patient knowledge or access to education that limit understanding of timely predialysis AV access and delay decision-making.
	Patient-related barriers (fear, refusal, misunderstanding)	Emotional or behavioral responses (e.g., fear, denial, refusal) that reduce willingness to pursue timely predialysis AV access preparation, despite information being available.
	Late CKD presentation	Patients presenting late in the CKD trajectory, leaving limited time for AV access preparation and shared decision-making.
	Establish support networks for patients and families	Suggestions to create peer, family, or community support systems (e.g., support groups or navigator-style support) to improve readiness.
	Increase accessible learning materials for patients	Calls for patient-friendly education materials (e.g., leaflets, videos, checklists) that are easy to access and understand.
	Provide multi-format public education	Broader public or community education campaigns across multiple media formats to raise awareness of CKD and predialysis AV access preparation.
**Theme 2. Care Pathway Coordination**	Multidisciplinary team collaboration	Interdisciplinary teamwork and alignment among nephrology, surgery, nursing, dialysis units, and other stakeholders.

	Referral network and collaboration between hospitals	Cross-institution referral pathways, shared protocols, and collaboration across sending and receiving facilities.
	Unclear or inefficient referral flow	Process gaps, unclear criteria, delays, or bottlenecks in referral and consultation workflows.
	Poor coordination among surgeons, nephrologists, and nurses	Communication failures and misalignment across roles resulting in missed timing for planning and follow-up.
	Lack of specialists or consulting network	Limited access to specialist consultation or a consulting network needed to coordinate evaluation and decision-making.
	Insufficient CKD knowledge among nurses & staff	Perceived gaps in staff knowledge about CKD staging, timing triggers, AV access preparation and patient counseling.
	Early CKD detection, CKD clinic, and timely referral for AV access planning within the ESKD life-plan framework	Early identification and structured CKD clinic follow-up enabling timely counseling, evaluation, and referral for access planning.
	Establish a dedicated AV access coordinator or coordination team	Suggestions to establish a dedicated coordinator-led team responsible for counseling, evaluation, scheduling, and tracking predialysis AV access preparation.
	Improve communication and coordination systems	Calls for improved communication and referral systems (e.g., shared protocols or electronic tracking) to support timely AV access counseling, evaluation, and procedures.
**Theme 3. System Capacity and Access**	Improved accessibility and reduced waiting time for AV access procedures	Need to reduce waiting time and improve access to AV access creation services, including appointments, operation-room availability, and streamlined scheduling.
	System delays / long consultation time	Operational delays in clinic or hospital processes, including consultations, investigations, and

		approvals, that postpone AV access planning and procedural scheduling
	Limited access to vascular surgeons	Insufficient availability of vascular surgeons or access providers leading to delays or limited service coverage.
	Adequate surgeons, operating-room time, and equipment for AV access procedures	Sufficient staffing, operating-room time, instruments, imaging or assessment tools, and procedural capacity to deliver timely AV access procedures.
	Increase specialist manpower	Recommendations to expand the specialist workforce (surgeons/interventionists) to meet demand and reduce queues.
**Theme 4. Policy and Financial Support**	Financial support and coverage for AV access procedures	Statements highlighting the role of coverage/benefits in enabling AV access creation and reducing out-of-pocket burden.
	Insufficient financial support	Financial barriers including uncovered procedures, travel/indirect costs, and inadequate support mechanisms.
	Policy and central-level support for proactive, stage-aligned AV access planning within the ESKD Life-Plan framework	Macro-level policy direction, central support, reimbursement rules, and system-level incentives shaping practice patterns.
**Theme 5. Context-specific Technical/ Clinical Suitability Considerations**	Other context-specific technical or clinical considerations	Setting-specific clinical or technical factors, such as vascular anatomy, comorbidity complexity, or local service capability, that influence the feasibility or appropriateness of timely predialysis AV access preparation but are not captured in Themes 1–4.
	Provider-suggested technical or clinical actions	Provider-suggested actions related to technical readiness, clinical complexity, or assessment resources that are context-specific and not captured in other themes.
