# Supplementary material for: Multidisciplinary providers’ perspectives on timely predialysis arteriovenous access preparation: An exploratory mixed-methods study in upper Northern Thailand
Source: PLoS One. 2026 Oct 5;21(10):e0359863. doi: 10.1371/journal.pone.0359863 (PMC13637856; doi:10.1371/journal.pone.0359863)
Supplement: Supplementary file 3 [file pone.0359863.s003.pdf]

Supporting Information

S3 File. Full list of quantitative items and descriptive statistics supporting Tables 3–5

This supporting Information file provides the complete item-level quantitative results from the provider questionnaires and explains the analytic rationale for selecting items presented in the joint displays in the main manuscript **(Tables 3–5).** Items were selected to illustrate analytically relevant patterns for mixed-methods integration, rather than to exhaustively represent or rank all measured questionnaire items. Qualitative excerpts in the joint displays were used to contextualize quantitative patterns and were not intended to indicate prevalence, dominance, relative importance, or statistical weighting. Providing the complete quantitative results alongside the selection rationale enhances the transparency and interpretability of the integrated findings.

There were no missing responses for the quantitative Likert-scale core barrier, profession-specific obstacle, or supportive-factor items; non-response occurred only for some open-ended questions and did not affect the quantitative item-level analyses.

Domain-level summaries are provided for descriptive overview only. Domain scores were calculated at the participant level by averaging item responses within each predefined domain and were then summarized by professional group using medians and interquartile ranges. Because Likert-scale responses are ordinal and the questionnaire was newly developed, the primary interpretation of quantitative findings was conducted at the item level. Domain-level scores were not used to select items for the main joint displays or to support confirmatory inference.

S3-A. Core barrier items and selection rationale for Table 3

Core barrier items were organized by a priori questionnaire domains. Item-level medians and interquartile ranges (IQRs) by professional role are reported in **Supplementary Table S3-A**.

Items included in **Table 3** were selected to illustrate analytically salient, cross-role barriers, rather than to comprehensively represent all measured items. Selection was based on the following predefined criteria:

1. A median rating ≥4.0 in at least two professional groups;
2. Relevance to key stages of the timely predialysis AV access care pathway; and
3. Availability of qualitative data to explain the observed quantitative pattern.

Accordingly, patient understanding and acceptance, late presentation or advanced CKD stage at first consultation, procedural waiting time for AV access, and insurance coverage and reimbursement processes were included. *Comorbidity affecting surgical suitability for AV access* were included as a prespecified analytic exception. Although this item did not meet the strict cross-role median criterion, it was prominent in the qualitative responses as a context-specific clinical consideration relevant to timely predialysis AV access preparation. Other barrier items were not selected for the joint display because they did not meet the analytic criteria or were conceptually redundant; however, they remain available in the complete supplementary results for transparency.

Supplementary Table S3-A. Full quantitative assessment of barriers to timely predialysis AV access preparation across professional roles

Domain	Item	Nurse (n=78)	Surgeon (n=19)	Nephrologist (n=16)	p
**Patient-level (5 items)**					
	Patient understanding and acceptance of timely predialysis AV access planning	4.0 (3.0, 5.0)	3.0 (2.0, 4.0)	4.0 (4.0, 4.5)	**0.016**
	Late presentation or advanced CKD stage at first consultation	4.0 (4.0, 5.0)	4.0 (3.4, 5.0)	4.0 (3.0, 4.0)	**0.016**
	Financial and travel constraints	4.0 (4.0, 5.0)	3.0 (3.0, 4.0)	3.5 (2.5, 4.0)	**<0.001**
	Comorbidities affecting clinical suitability for AV access	4.0 (3.0, 4.0)	3.0 (3.0, 4.0)	2.5 (2.0, 3.0)	**<0.001**
	Adherence and reliability of follow-up	3.0 (3.0, 4.0)	3.0 (2.0, 4.0)	2.5 (2.0, 3.0)	**0.012**
**Provider/team-level (1 item)**					
	Coordination among multidisciplinary team members (nurses, nephrologists, and surgeons)	3.0 (2.0, 4.0)	3.0 (3.0, 4.0)	2.0 (1.5, 3.0)	**0.006**
**System-level (4 items)**					
	Procedural waiting time for AV access	4.0 (3.0, 5.0)	3.0 (2.0, 4.0)	4.0 (2.0, 4.5)	**0.030**
	Insurance coverage and reimbursement processes	4.0 (3.0, 5.0)	4.0 (3.0, 4.0)	3.5 (2.0, 5.0)	0.342
	Availability of surgical resources (operating room, personnel, and equipment)	4.0 (3.0, 5.0)	3.0 (1.0, 4.0)	3.5 (3.0, 4.0)	**<0.001**
	Efficiency of referral and follow-up systems	4.0 (3.0, 4.0)	3.0 (2.0, 4.0)	3.5 (2.7, 3.6)	**0.019**
**Domain Scores**					
	Patient-level domain (5 items)	4.0 (3.6, 4.2)	3.4 (3.0, 3.8)	3.3 (2.9, 3.4)	**<0.001**

	Provider-level domain (1 item)	3.0 (2.0, 4.0)	3.0 (3.0, 4.0)	2.0 (1.5, 3.0)	**0.002**
	System-level domain (4 items)	3.8 (3.2, 4.2)	3.2 (2.2, 3.5)	3.5 (2.6, 3.6)	**0.003**
	Global obstacle score (10 items)	3.8 (3.3, 4.1)	3.4 (2.8, 3.6)	3.2 (2.6, 3.3)	**<0.001**

Notes: Values are presented as median (Q1, Q3). Barrier items were rated on 5-point Likert scales (1 = not a barrier to 5 = very major barrier). Because the Likert-scale items were treated as ordinal, results are summarized using median and interquartile range (IQR), and group comparisons were conducted using Kruskal–Wallis tests. P-values were not adjusted for multiple comparisons because the quantitative analyses were exploratory and intended to describe response patterns for mixed-methods integration rather than to test confirmatory hypotheses. Domain-level summaries represent the median (IQR) of participant-level domain scores calculated as the mean of item scores within each predefined domain.

S3-B. Role-Specific Obstacle Items and Selection Rationale for Table 4

Role-specific obstacle items were assessed using profession-tailored questionnaires and are reported in full in **Supplementary Table S3-B**.

Table 4 was designed to illustrate within-role workflow constraints, rather than cross-role comparison. For each professional group, the single role-specific item with the highest median score was selected. When multiple items shared the same median, selection was guided by analytic representativeness, prioritizing items that best reflected the core responsibilities and decision-making role of that profession within the timely predialysis AV access care pathway.

Accordingly, the ability to support patient and family self-care and readiness was selected for nurses, the technical complexity of AV access procedures in high-risk or complex cases for vascular surgeons, and the preparation of patients prior to AV access planning and creation (medical and psychological) for nephrologists. Qualitative excerpts were selected to align directly with the chosen quantitative items and serve an explanatory function.

Supplementary Table S3-B. Full quantitative assessment of profession-specific obstacle items

**Domain**	**Item**	**Median (IQR)**
**A. Nurse (n=78)**		
Patient-lavel	1. Effectiveness of patient education and AV access preparation programs	3.0 (2.0, 4.0)
	2. Ability to support patient and family self-care and readiness	3.0 (3.0, 4.0)
	Patient-role domain score (2 items)	3.0 (2.5, 4.0)
Provider-level	3. Coordination within the multidisciplinary team during patient follow-up	3.0 (2.0, 4.0)
	4. Competency in assessing AV access maturation prior to cannulation	3.0 (2.0, 4.0)
	Provider-role domain score (2 items)	3.0 (2.5, 3.5)
**B. Surgeon (n=19)**		
Provider-level	1. Coordination with referring nephrologists	3.0 (2.0, 4.0)
	2. System for post-op complications monitoring and management	3.0 (2.0, 4.0)
	3. Technical complexity of AV access procedures in high-risk or complex cases	3.0 (2.0, 4.0)

	Provider-role domain score (3 items)	2.7 (2.2, 3.7)
System-level	4. Operating theatre scheduling and resource management	3.0 (2.0, 4.0)
	5. Preoperative vascular assessment and vessel quality evalution	3.0 (2.0, 4.0)
	System-role domain score (2 items)	3.0 (2.0, 3.5)
**C. Nephrologist (n=16)**		
Provider-level	1. Clarity of criteria for selecting candidates for timely predialysis AV access	2.0 (1.0, 2.5)
	2. Backup plan when AV access creation (e.g., AVF or AVG) is not feasible or unsuccessful	2.0 (2.0, 3.0)
	3. Determination of optimal timing for surgical referral	3.0 (2.0, 3.5)
	4. Preparation of patients prior to AV access planning and creation (medical and psychological)	3.5 (2.0, 4.0)
	Provider-role domain score (4 items)	2.4 (2.0, 3.0)
System-level	5. Burden of monitoring CKD progression and predicting dialysis initiation	2.0 (2.0, 3.0)
	System-role domain score (1 item) [1]	2.0 (2.0, 3.0)

Notes: Values are presented as median (Q1, Q3). Items were rated on 5-point Likert scales (1 = not a barrier to 5 = very major barrier). For each professional group, domain-level summaries represent the median (IQR) of participant-level domain scores calculated as the mean of item scores within each domain. Role-specific obstacle items were administered only to the corresponding provider group; therefore, no statistical comparisons across professions were performed. The system-role domain for nephrologists comprises a single item assessing the burden of CKD monitoring.

S3-C. Supportive factor items and selection rationale for Table 5

Supportive-factor items were grouped into the care coordination, system capacity and training resources, and policy and financial support domains and are reported in full in **Supplementary Table S3-C**.

Items included in **Table 5** were defined as convergent supportive factors and met the predefined criterion of a median rating ≥4.0 among nurses, vascular surgeons, and nephrologists. Among the eligible items, final selection required relevant qualitative material that could provide contextual explanation or provider-suggested actions.

Based on these criteria, the presence of a dedicated vascular access coordinator, electronic referral and longitudinal follow-up systems, predictable financial coverage and reimbursement mechanisms for AV access procedures, and proactive, stage-aligned AV access planning guidelines within the ESKD Life-Plan framework were included. Other supportive factors meeting the quantitative threshold were not included in the joint display to reduce redundancy and maintain analytic focus; their complete results are reported in **Supplementary Table S3-C**.

Supplementary Table S3-C. Full quantitative assessment of supportive factors related to timely predialysis AV access preparation across professional roles

Domain	Item	Nurse (n=78)	Surgeon (n=19)	Nephrologist (n=16)	p
Care coordination	Presence of a dedicated vascular access coordinator	4.0 (4.0, 5.0)	4.0 (3.0, 4.0)	4.0 (4.0, 5.0)	**0.002**
	Inter-institutional collaboration and referral network	4.0 (4.0, 5.0)	4.0 (3.0, 4.0)	4.0 (3.0, 4.0)	**<0.001**

System capacity and training resources	Electronic referral and longitudinal follow-up system	4.0 (4.0, 5.0)	4.0 (4.0, 4.0)	4.0 (3.0, 4.0)	**0.004**
	Ongoing training program related to AV access care	4.0 (4.0, 5.0)	4.0 (4.0, 4.0)	4.0 (3.0, 4.0)	**<0.001**
Policy and financial support	Predictable financial coverage and reimbursement mechanisms for AV access procedures	5.0 (4.0, 5.0)	4.0 (3.0, 4.0)	4.5 (4.0, 5.0)	**0.005**
	Proactive, stage-aligned AV access planning guidelines within the ESKD Life-Plan framework	5.0 (4.0, 5.0)	4.0 (4.0, 4.5)	4.5 (4.0, 5.0)	0.076
Domain scores	Care coordination domain (2 items)	4.0 (4.0, 5.0)	4.0 (3.5, 4.0)	4.0 (3.5, 4.5)	**<0.001**
	System capacity and training resources domain (2 items)	4.2 (4.0, 5.0)	4.0 (4.0, 4.0)	3.8 (3.2, 4.0)	**<0.001**
	Policy and financial support domain (2 items)	4.5 (4.0, 5.0)	4.0 (3.5, 4.5)	4.5 (3.8, 5.0)	**0.016**
	Overall supportive-factor score (6 items)	4.3 (4.0, 5.0)	4.0 (3.7, 4.3)	4.2 (3.6, 4.3)	**<0.001**

Notes: Supportive factors were rated on 5-point Likert scales (1 = no role to 5 = very important role). Values are presented as median (Q1, Q3). Domain-level summaries represent the median (IQR) of participant-level domain scores, calculated as the mean of item scores within each domain; the overall supportive-factor score represents the participant-level mean of all six supportive-factor items. Comparisons across professional groups were performed using Kruskal–Wallis test. There are no missing responses for the supportive-factor items. P-values were not adjusted for multiple comparisons because the analyses were exploratory and intended to describe response patterns for mixed-methods integration rather than to test confirmatory hypotheses.
