# Supplementary material for: Multidisciplinary providers’ perspectives on timely predialysis arteriovenous access preparation: An exploratory mixed-methods study in upper Northern Thailand
Source: PLoS One. 2026 Oct 5;21(10):e0359863. doi: 10.1371/journal.pone.0359863 (PMC13637856; doi:10.1371/journal.pone.0359863)
Supplement: Supplementary file 4 [file pone.0359863.s004.pdf]

Supporting Information

S4 File. Reflexivity and analytic rigor statement

The research team comprised clinicians and health services researchers with experience in nephrology care, vascular access planning, and multidisciplinary service delivery within the Thai health system. The team also included a researcher with expertise in mixed-methods research and nursing care for people with ESKD. This expertise informed the review of qualitative themes, mixed-methods integration, and interpretation of nursing perspectives. Recognizing that professional and disciplinary familiarity with these care pathways could influence interpretation, reflexive practices were applied throughout the analysis to critically examine assumptions related to barriers and supportive factors associated with timely predialysis AV access preparation.

Qualitative analysis followed a combined deductive-inductive, team-based approach. Initial codes were developed from the open-ended responses. Given the brief written format, the analysis focused on identifying recurring concepts and providing contextual detail rather than developing an in-depth interpretive theory. Code and themes were refined iteratively and permitted to map to multiple themes when conceptually relevant, reflecting the interconnected nature of real-world care pathways. Representative quotations were selected to illustrate analytic patterns rather than to indicate prevalence, dominance, or relative importance, as documented in the qualitative coding framework (**S2 File**).

Mixed-methods integration was conducted using a convergent approach. Quantitative data were used to identify ratting patterns across professional roles, while qualitative data provided context for how providers understood and described the selected barriers, role-specific obstacles, and supportive factors. Joint displays (**Tables 3–5**) were constructed based on conceptual relevance rather than structural equivalence between quantitative domains and qualitative themes. The joint displays were illustrative rather than exhaustive and were not intended to rank findings by prevalence or importance. Complete quantitative item-level results and selection rationales are provided in **S3 File** to support transparency while preserving analytic focus.
