# Supplementary material for: Multidisciplinary providers’ perspectives on timely predialysis arteriovenous access preparation: An exploratory mixed-methods study in upper Northern Thailand
Source: PLoS One. 2026 Oct 5;21(10):e0359863. doi: 10.1371/journal.pone.0359863 (PMC13637856; doi:10.1371/journal.pone.0359863)
Supplement: Supplementary file 6 [file pone.0359863.s007.docx]

**Second revision**

**PONE-D-26-17428R1**

**Response to editor and reviewers**

**Journal:** PLOS ONE

**Manuscript ID:** PONE-D-26-17428R1

**Title:** Multidisciplinary Providers’ Perspectives on Timely Arteriovenous Access Planning Prior to Hemodialysis: An Exploratory Mixed-Methods Study in Upper Northern Thailand

**Corresponding author:** Kittipan Rerkasem, M.D.

**Date:** 9 Sep 2026

Dear Dr. Phannajit and Reviewers,

Thank you for the careful evaluation of our revised manuscript and for the constructive comments. We have addressed each point raised by the Academic Editor and Reviewer #2 and have carefully checked the manuscript for language, terminology, references, and internal consistency. Reviewer comments are reproduced below, followed by our point-by-point responses. Changes are shown in the marked-up manuscript and incorporated into the clean revised manuscript.

We hope that the revised manuscript is now suitable for publication in PLOS ONE.

Sincerely,

Kittipan Rerkasem, M.D.
On behalf of all authors

# Response to the Academic Editor

**Comment:** Please carefully recheck the grammatical errors and terms consistency as stated by reviewer 2.

**Response:** Thank you for this guidance. We carefully proofread the entire manuscript and corrected the remaining grammatical, typographical, and wording errors. We also performed a final terminology consistency check across the main text, tables, Figure 1, and S1–S4 Files. The terminology is now used consistently according to the distinctions described in our responses to Reviewer #2. All revisions are shown in the marked-up files and do not alter the study findings.

# Response to Reviewer #1

**Comment:** Thank you to the authors for your thorough and thoughtful revisions. The authors have addressed all of my comments clearly and incorporated the necessary modifications into the manuscript. I am fully satisfied with the responses.

**Response:** We sincerely thank the reviewer for the careful evaluation of our revised manuscript and for confirming that the previous comments have been adequately addressed. We greatly appreciate the reviewer’s constructive feedback, which has helped us improve the clarity and quality of the manuscript.

# Response to Reviewer #2

We sincerely thank Reviewer #2 for the careful reassessment of our manuscript and for recognizing the substantive improvements made during the previous revision. We have addressed each remaining editorial and consistency issue below.

**Comment 1.** Please undertake a careful final English-language proofread of the entire manuscript. Several grammatical and wording errors remain, including subject–verb agreement, pluralization, duplicated wording, and typographical errors. Examples include “AV access preparation remain suboptimal,” “Integrated finding identified,” “in many setting,” “For supportive factors received,” “these ratting patterns,” and “Across professional groups, provider shared.” These issues do not affect the scientific conclusions but should be corrected to improve clarity and readability.

**Response:** Thank you for identifying these language and wording issues. We carefully proofread the entire manuscript and corrected errors in subject–verb agreement, pluralization, sentence construction, word choice, spelling, punctuation, duplicated wording, and typographical presentation. Specifically, “AV access preparation remain suboptimal” was corrected to “AV access preparation remains suboptimal”; “Integrated finding identified” was revised to “Integration of the quantitative and qualitative findings identified”; “in many setting” was corrected to “in many settings”; “For supportive factors received” was corrected to “Four supportive factors received”; “these ratting patterns” was corrected to “these rating patterns”; and “Across professional groups, provider shared” was corrected to “Across professional groups, providers shared.”

We also corrected additional language and wording issues throughout the manuscript to improve clarity, readability, and consistency. These editorial revisions did not alter the study data, analyses, results, or interpretation.

**Changes in the manuscript:** Corrections were made throughout the Abstract, Introduction, Methods, Results, Discussion, Conclusion, tables, and Figure 1 caption and are shown in the marked-up manuscript.

**Comment 2.** Please correct the incomplete sentence in the Results section describing insurance and reimbursement barriers. The current text ends with “qualitative responses referring to coverage limitations and administrative” before immediately proceeding to the sentence concerning comorbidity and clinical suitability. The missing wording should be restored so that the paragraph is grammatically complete and its intended meaning is clear.

**Response:** Thank you for identifying this incomplete sentence. The final portion of the sentence was inadvertently omitted during the previous revision. We have restored the missing wording and corrected the duplicated expression “barriers constraints.” The revised sentence now reads: *“Insurance coverage and reimbursement processes received median ratings of 4.0 among nurses and surgeons and 3.5 among nephrologists, with qualitative responses referring to coverage limitations and administrative delays.”* The paragraph is now grammatically complete and clearly conveys the intended finding.

**Changes in the manuscript:** Results, subsection “Integrated barriers to timely predialysis AV access preparation,” page 16, lines 436–438.

**Comment 3.** Please perform a final consistency check of terminology throughout the manuscript. Terms such as “predialysis AV access preparation,” “planning and creation,” “supportive factors,” “supportive strategies,” and “facilitators” are sometimes used interchangeably. Complete uniformity is not necessary where the concepts genuinely differ, but terminology should be used consistently within the Abstract, Results, Discussion, tables, figure, and Conclusion.

**Response:** Thank you for this helpful comment. We conducted a final terminology review across the Abstract, main text, tables, Figure 1, Conclusion, and Supporting Information. We now use *“predialysis AV access preparation”* as the umbrella term encompassing planning and, when clinically appropriate, surgical creation of an AVF or AVG before hemodialysis initiation. *“Planning and creation”* is retained only where the text specifically refers to these distinct stages of preparation.

We use *“supportive factors”* for the six quantitative questionnaire items, *“supportive strategies”* when discussing the four consistently highly rated factors as candidate components for future service evaluation, and *“provider-suggested actions”* for recommendations derived from the open-ended responses. *“Facilitators”* is no longer used as an analytic label in the main manuscript and is retained only in S1 File, where it reproduces the wording of the original open-ended questionnaire item.

Relevant headings, table labels, captions, notes, Figure 1, and Supporting Information files were revised accordingly. These changes improve terminological consistency without altering the study data, analyses, or findings.

**Changes in the manuscript:** Terminology was standardized throughout the Abstract, Introduction, Methods, Results, Discussion, Conclusion, Tables 1–5, Figure 1 and its caption, and S1–S4 Files, as shown in the marked-up files.

**Comment 4.** Please carefully recheck the reference list against the original publications. There appear to be remaining bibliographic and formatting inconsistencies, including misplaced or potentially incorrect DOI information and irregular formatting of some references. For example, the entries for Xi et al., Allon et al., and Sachdeva and Abreo should be verified carefully. Journal names, punctuation, author formatting, article titles, volume/page information, and DOI assignments should all be checked before final acceptance.

**Response:** Thank you for drawing our attention to these bibliographic inconsistencies. We carefully rechecked the entire reference list against the original publications and corrected the journal names, punctuation, author formatting, article titles, publication years, volume and page information, and DOI assignments, as appropriate.

In particular, the DOI for Xi et al., which had been inadvertently placed under the Allon et al. entry, was reassigned to the correct reference. The Allon et al. entry was updated with its correct DOI and standardized bibliographic information. The Sachdeva and Abreo entry was also corrected to remove duplicated journal information and irregular punctuation and to standardize the author and article-title formatting. Additional inconsistencies elsewhere in the reference list were corrected to ensure a uniform bibliographic format.

**Comment 5.** Please conduct one final editorial check of tables, Supporting Information citations, and figure references. The revised manuscript has substantially improved the transparency of the joint-display selection criteria and the distinction between quantitative findings and qualitative contextualization. The authors should ensure that references to S1–S4 Files, Tables 1–5, and Figure 1 are internally consistent and that all captions and notes use the final terminology adopted in the main text.

**Response:** Thank you for this helpful comment. We conducted a final consistency check of Tables 1–5, Figure 1, and S1–S4 Files. All citations, captions, labels, and notes were corrected and harmonized with the terminology used in the main text. Quantitative findings, qualitative contextualization, and provider-suggested actions are now clearly distinguished throughout.

**Changes in the manuscript and Supporting Information:** Relevant cross-references and terminology were standardized throughout the marked-up manuscript, Figure 1, and S1–S4 Files.
