# Supplementary material for: Multidisciplinary providers’ perspectives on timely predialysis arteriovenous access preparation: An exploratory mixed-methods study in upper Northern Thailand
Source: PLoS One. 2026 Oct 5;21(10):e0359863. doi: 10.1371/journal.pone.0359863 (PMC13637856; doi:10.1371/journal.pone.0359863)
Supplement: Peer review history

# Peer review: Decision Letter 0

**DOI:** 10.1371/journal.pone.0359863.r001

**Authors:** Jeerath Phannajit

5 Jul 2026

Dear Dr. Rerkasem,

Thank you for submitting your manuscript to PLOS ONE. After careful consideration, we feel that it has merit but does not fully meet PLOS ONE’s publication criteria as it currently stands. Therefore, we invite you to submit a revised version of the manuscript that addresses the points raised during the review process.

Please submit your revised manuscript by Aug 19 2026 11:59PM. If you will need more time than this to complete your revisions, please reply to this message or contact the journal office at plosone@plos.org. When you're ready to submit your revision, log on to https://www.editorialmanager.com/pone/ and select the 'Submissions Needing Revision' folder to locate your manuscript file.

- A letter that responds to each point raised by the academic editor and reviewer(s). You should upload this letter as a separate file labeled 'Response to Reviewers'.
- A marked-up copy of your manuscript that highlights changes made to the original version. You should upload this as a separate file labeled 'Revised Manuscript with Track Changes'.
- An unmarked version of your revised paper without tracked changes. You should upload this as a separate file labeled 'Manuscript'.

If you would like to make changes to your financial disclosure, please include your updated statement in your cover letter. Guidelines for resubmitting your figure files are available below the reviewer comments at the end of this letter.

If applicable, we recommend that you deposit your laboratory protocols in protocols.io to enhance the reproducibility of your results. Protocols.io assigns your protocol its own identifier (DOI) so that it can be cited independently in the future. For instructions see: https://journals.plos.org/plosone/s/submission-guidelines#loc-laboratory-protocols. Additionally, PLOS ONE offers an option for publishing peer-reviewed Lab Protocol articles, which describe protocols hosted on protocols.io. Read more information on sharing protocols at https://plos.org/protocols?utm_medium=editorial-email&utm_source=authorletters&utm_campaign=protocols.

As the corresponding author, your ORCID iD is verified in the submission system and will appear in the published article. PLOS supports the use of ORCID, and we encourage all coauthors to register for an ORCID iD and use it as well. Please encourage your coauthors to verify their ORCID iD within the submission system before final acceptance, as unverified ORCID iDs will not appear in the published article. *Only*  the individual author can complete the verification step; PLOS staff *cannot*  verify ORCID iDs on behalf of authors.

We look forward to receiving your revised manuscript.

Kind regards,

Jeerath Phannajit, M.D, Ph.D.

Academic Editor

PLOS One

Journal Requirements:

When submitting your revision, we need you to address these additional requirements.

1. Please ensure that your manuscript meets PLOS ONE's style requirements, including those for file naming. The PLOS ONE style templates can be found at

https://journals.plos.org/plosone/s/file?id=wjVg/PLOSOne_formatting_sample_main_body.pdf and

https://journals.plos.org/plosone/s/file?id=ba62/PLOSOne_formatting_sample_title_authors_affiliations.pdf.

2. Thank you for stating in your Funding Statement:

“This study was supported by the Health Systems Research Institute (HSRI), Thailand (Grant No. HSRI.67-180), and Chiang Mai University. The funders had no role in study design, data collection and analysis, decision to publish, or preparation of the manuscript. “

Please provide an amended statement that declares *all* the funding or sources of support (whether external or internal to your organization) received during this study, as detailed online in our guide for authors at http://journals.plos.org/plosone/s/submit-now.  Please also include the statement “There was no additional external funding received for this study.” in your updated Funding Statement.

Please include your amended Funding Statement within your cover letter. We will change the online submission form on your behalf.

3. Thank you for stating the following in the Acknowledgments Section of your manuscript:

“This study was supported by the Health Systems Research Institute (HSRI), Thailand (Grant No. HSRI.67-180), and Chiang Mai University. The funders had no role in study design, data collection and analysis, decision to publish, or preparation of the manuscript.”

We note that you have provided additional information within the Acknowledgements Section that is not currently declared in your Funding Statement. Please note that funding information should not appear in the Acknowledgments section or other areas of your manuscript. We will only publish funding information present in the Funding Statement section of the online submission form.

Please remove any funding-related text from the manuscript and let us know how you would like to update your Funding Statement. Currently, your Funding Statement reads as follows:

“This study was supported by the Health Systems Research Institute (HSRI), Thailand (Grant No. HSRI.67-180), and Chiang Mai University. The funders had no role in study design, data collection and analysis, decision to publish, or preparation of the manuscript.”

Please include your amended statements within your cover letter; we will change the online submission form on your behalf.

4. We note that your Data Availability Statement is currently as follows: [All relevant data are within the manuscript and its Supporting Information files. ]

Please confirm at this time whether or not your submission contains all raw data required to replicate the results of your study. Authors must share the “minimal data set” for their submission. PLOS defines the minimal data set to consist of the data required to replicate all study findings reported in the article, as well as related metadata and methods (https://journals.plos.org/plosone/s/data-availability#loc-minimal-data-set-definition).

For example, authors should submit the following data:

- The values behind the means, standard deviations and other measures reported;

- The values used to build graphs;

- The points extracted from images for analysis.

Authors do not need to submit their entire data set if only a portion of the data was used in the reported study.

If your submission does not contain these data, please either upload them as Supporting Information files or deposit them to a stable, public repository and provide us with the relevant URLs, DOIs, or accession numbers. For a list of recommended repositories, please see https://journals.plos.org/plosone/s/recommended-repositories.

If there are ethical or legal restrictions on sharing a de-identified data set, please explain them in detail (e.g., data contain potentially sensitive information, data are owned by a third-party organization, etc.) and who has imposed them (e.g., an ethics committee). Please also provide contact information for a data access committee, ethics committee, or other institutional body to which data requests may be sent. If data are owned by a third party, please indicate how others may request data access.

5. If the reviewer comments include a recommendation to cite specific previously published works, please review and evaluate these publications to determine whether they are relevant and should be cited. There is no requirement to cite these works unless the editor has indicated otherwise.

Additional Editor Comments (if provided):

Please revise the manuscript to clearly present the study as an exploratory, regional, provider-perception study rather than a definitive assessment of determinants of delayed AV access creation. Please address the following key points:

1. Revise the abstract, results, discussion, and conclusion to avoid overgeneralization. The findings should be framed as perceived barriers and facilitators among providers in Upper Northern Thailand, not as nationally representative or causal findings.
2. Clarify the sampling strategy and its limitations, including recruitment through an academic meeting/professional networks, possible selection bias, small numbers of nephrologists and vascular surgeons, and the predominance of dialysis nurses.
3. Address the concern that dialysis nurses may provide mainly retrospective views of predialysis care. Please clarify whether any nurses had active CKD stage 4–5 or predialysis access-planning roles. If CKD/predialysis nurses were not included, this should be acknowledged as a limitation.
4. Clarify that patient-level barriers reflect provider perceptions only. The absence of direct patient perspectives and objective clinical/system data, such as catheter use at initiation, AVF/AVG creation rates, referral-to-surgery time, surgical wait time, and reimbursement delays, should be explicitly discussed.
5. Strengthen the description of questionnaire development and validation, including item generation, expert review, pilot testing, item revision, and interpretation of Cronbach’s alpha.
6. Clarify the statistical analysis, including missing data, p-values, multiple comparisons, use of domain-level Likert scores, and the absence of a formal sample-size calculation. Cross-professional comparisons should be presented as exploratory.
7. Clarify the mixed-methods component. Please state the nature and depth of the qualitative data, the number of open-ended responses, coding procedures, and how qualitative and quantitative findings were integrated.
8. Ensure transparent reporting of selected results. If Tables 3–5 present selected items, please provide complete item-level results in the supplementary materials and describe the joint displays as illustrative.
9. Revise Figure 1 to improve conceptual consistency. In particular, “procedural wait time” appears to be a system-level barrier rather than a patient/family readiness factor.
10. Revise Table 1 to improve workload reporting across professional groups. If nephrologists’ active dialysis patient workload was not collected, please state this.
11. Please also shorten and focus the Discussion, reduce repetitive conceptual language, and distinguish clearly between observed findings, interpretation, and hypotheses for future service improvement.

[Note: HTML markup is below. Please do not edit.]

Reviewers' comments:

Reviewer's Responses to Questions

**Comments to the Author**

1. Is the manuscript technically sound, and do the data support the conclusions?

Reviewer #1: Partly

Reviewer #2: Yes

**********

2. Has the statistical analysis been performed appropriately and rigorously?

Reviewer #1: Yes

Reviewer #2: Yes

**********

3. Have the authors made all data underlying the findings in their manuscript fully available?

The PLOS Data policy

Reviewer #1: Yes

Reviewer #2: Yes

**********

4. Is the manuscript presented in an intelligible fashion and written in standard English?

Reviewer #1: No

Reviewer #2: Yes

**********

Reviewer #1: The idea of conducting this study is practically reasonable. However, after reviewing the article, I have the following comments.

1. Methodological Misalignment in Sample Selection: A major limitation of this study is the heavy reliance on dialysis nurses, who constitute 69% of the total sample, to evaluate predialysis vascular access planning. Dialysis nurses primarily care for patients who have already initiated hemodialysis, meaning their insights into predialysis barriers are retrospective and subject to recall bias. The study notably omits the perspectives of Chronic Kidney Disease (CKD) or predialysis nurses, who are actively responsible for patient education, tracking readiness, and coordinating care during CKD stages 4 and 5. Consequently, the dominant nursing voice in this paper reflects post-dialysis observations rather than active, frontline management of the predialysis pathway, potentially skewing the validity of the identified workflow constraints.

2. Psychometric Instability Linked to Proxy Reporting: The suboptimal internal consistency of the patient-level domain (Cronbach's alpha = 0.67) serves as direct statistical evidence of a fundamental design flaw: attempting to measure patient-level constructs through proxy provider perspectives. Because healthcare providers—particularly dialysis nurses who only manage patients retrospectively—must speculate on the behavioral and educational barriers of the predialysis phase, their ratings might be suboptimal. This mismatch causes high variance and poor item-to-item correlation within the subscale. Had the authors directly surveyed or interviewed the patients themselves, the items mapping onto patient readiness would likely have demonstrated significantly higher psychometric reliability and conceptual cohesion.

3. Suboptimal Definition of the Target Patient Phenomenon: A fundamental methodological weakness of this study is its broad, undifferentiated focus on all patients initiating dialysis without permanent access. A substantial portion of advanced CKD patients are clinically or anatomically unsuitable for AVF creation due to vascular exhaustion or severe comorbidities. To effectively isolate system-level and behavioral barriers to timely planning, the study should have utilized a targeted case-selection strategy. Specifically, it should have focused on the subset of patients who were clinically viable candidates for an AVF but still initiated dialysis via a catheter before subsequently undergoing successful AVF creation. Interviewing the specific providers managing this precise "delayed-but-eligible" cohort would have yielded highly actionable insights into care-pathway bottlenecks, rather than capturing generalized perceptions of a heterogeneous patient pool.

4. Inconsistent Workload Metrics and Data absence in Table 1: In Table 1, the authors fail to report the number of active dialysis patients managed per month by participating nephrologists, leaving a notable data gap in the descriptive statistics. While dialysis nurses' workloads are quantified by "dialysis patients/month", nephrologists are only evaluated on their predialysis CKD 4–5 volume. In clinical practice, particularly in Thailand, nephrologists actively manage and round on maintenance hemodialysis patients.

5. Conceptual Inconsistency and Poor Visual Flow in Figure 1: The conceptual model presented in Figure 1 contains significant logical contradictions and fails to establish a well-connected flow linking specific barriers to the stages of the predialysis care pathway. Notably, "Procedural wait time" is placed under the domain of "Patient & family readiness". This probably mischaracterizes a structural, system-level capacity barrier as a patient-level factor and directly contradicts the authors' own classification in Table 3, where it is correctly labeled as a system-level barrier. Furthermore, the duplicated labeling of "Patient & family readiness" boxes on the left side of the diagram reflects poor visual creation.

Reviewer #2: This manuscript addresses an important and clinically relevant issue: delayed predialysis arteriovenous access planning and creation before hemodialysis initiation in Thailand. The topic is worthwhile, and the attempt to compare perspectives among nephrologists, vascular surgeons, and dialysis nurses is potentially valuable. Understanding multidisciplinary barriers to timely AV access preparation is relevant for improving predialysis care pathways, reducing catheter dependence, and informing service planning.

However, the manuscript requires major revision before it can be considered further. The study has potential, but several important methodological, analytic, and reporting issues need to be addressed. In particular, the conclusions should be made more cautious and better aligned with the exploratory nature of the data.

1. The first major concern relates to the sampling strategy and representativeness. The study is described as a cross-sectional convergent mixed-methods study, but recruitment was pragmatic and opportunity-based, relying primarily on a regional vascular access academic meeting and professional networks. This approach is understandable for an exploratory study, but it introduces a substantial risk of selection bias. Providers attending such a meeting or reachable through professional networks may already be more engaged with vascular access planning than the broader provider population. The authors should discuss this limitation more explicitly and avoid implying that the findings represent all providers in Northern Thailand or Thailand as a whole.

2. The sample size and imbalance across professional groups also require more careful handling. Although the total sample included 113 participants, the subgroup sizes were uneven, with 78 nurses, 19 vascular surgeons, and only 16 nephrologists. These small numbers limit the reliability of cross-professional comparisons. The manuscript repeatedly emphasizes role-specific differences, workflow misalignment, and operational differences across professional groups. These observations may be useful, but they should be framed as exploratory and hypothesis-generating rather than definitive. The authors should revise the Results, Discussion, and Conclusion to avoid overinterpreting small subgroup differences.

3. The absence of a formal sample-size calculation should also be addressed more clearly. The authors state that the study was exploratory and not intended to test a predetermined effect size. This is acceptable, but the manuscript should then consistently present the quantitative findings as descriptive. The authors should avoid language suggesting strong comparative inference unless supported by appropriate statistical evidence.

4. The mixed-methods component needs clearer justification and more cautious interpretation. The manuscript presents the study as a convergent mixed-methods design, but the qualitative component appears to be based on brief open-ended survey responses rather than in-depth interviews or focus groups. Open-ended survey responses can provide useful contextual information, but they usually provide limited depth. Therefore, claims about underlying mechanisms, workflow dynamics, or operational misalignment should be softened. The authors should clarify the depth and nature of the qualitative data, report how many participants provided open-ended responses, and explain how coding reliability or analytic credibility was ensured.

5. The questionnaire development and validation process should be described in more detail. The manuscript states that exploratory interviews informed item development, followed by expert review and pilot testing. However, important details are missing, including how interview findings were translated into survey items, how content validity was assessed, how pilot testing was performed, whether items were revised after pilot testing, and whether the instrument was assessed for clarity across all three professional groups. Cronbach’s alpha values are helpful, but internal consistency alone does not establish validity, particularly for a newly developed instrument. The authors should strengthen this section and consider moving the full questionnaire and validation process into a clearly described supplementary file.

6. The statistical analysis also requires clarification. Likert-scale responses were summarized using medians and interquartile ranges and compared using Kruskal-Wallis tests, which is reasonable for exploratory analysis. However, the manuscript should more clearly report p-values, missing-data handling, and whether any adjustment for multiple comparisons was considered. If no adjustment was applied, this should be stated and justified. The authors should also consider reporting effect sizes or emphasizing descriptive patterns rather than statistical significance, given the small subgroup sizes.

7. The use of domain-level scores created by averaging Likert items should be justified. Because Likert responses are ordinal and the instrument is newly developed, the authors should explain why averaging items was appropriate and whether sensitivity analyses were performed using item-level responses only. If domain-level scores are not central to the interpretation, the authors may consider simplifying the analysis and focusing primarily on item-level descriptive findings.

8. The joint displays are useful, but the selection process may create concern about selective reporting. The manuscript states that Tables 3-5 include selected items based on predefined analytic criteria rather than all measured items. This approach can be acceptable in mixed-methods research, but the authors should ensure that the main manuscript provides sufficient information for readers to judge whether the selected items fairly represent the full dataset. The complete item-level results should be clearly provided in the supplementary materials, and the manuscript should explicitly state that the joint displays are illustrative rather than exhaustive.

9. The discussion and conceptual interpretation should be shortened and made more cautious. Terms such as “operational misalignment,” “workflow execution,” “role-specific expectations,” and “system-level operationalization” are repeated frequently. These concepts may be useful, but the study did not directly observe workflows, referral processes, reimbursement pathways, surgical scheduling, patient behavior, or actual AV access outcomes. The data primarily reflect provider perceptions. Therefore, the authors should revise the Discussion to distinguish clearly between observed survey findings, qualitative interpretations, and hypotheses about system-level mechanisms.

10. The manuscript would also benefit from clearer acknowledgment that objective clinical and system-level outcome data were not collected. The central issue is timely predialysis AV access planning and creation, but the study does not measure actual AVF/AVG creation rates, referral-to-surgery time, catheter use at dialysis initiation, failed referrals, surgical wait times, reimbursement delays, or patient-level reasons for noncompletion. This does not invalidate the study, but it limits what can be concluded. The authors should present their findings as perceived barriers and facilitators rather than determinants of actual delayed access creation.

11. The title, abstract, and conclusion should be revised to avoid overgeneralization. Because the study was conducted in Upper Northern Thailand, the authors should avoid phrasing that implies national representativeness. The abstract should clearly state the regional setting and exploratory nature of the study. The conclusion should emphasize that the findings provide preliminary, context-specific insights that may inform future service improvement work, rather than implying that the proposed strategies have been proven effective.

12. The writing is generally clear, but the manuscript is repetitive and could be substantially shortened. Phrases such as “predialysis AV access planning and creation,” “system-level strategies,” “role-specific interpretations,” “workflow constraints,” and “operational misalignment” recur throughout the manuscript. The Discussion in particular would benefit from consolidation. The authors should reduce repeated conceptual language and more directly link each interpretation to specific findings.

13. Finally, the reference list should be carefully checked before publication. Most references appear relevant, but several entries may require correction of author formatting, publication year, article type, DOI, or bibliographic details. The authors should verify all references against PubMed, Crossref, ThaiJO, and publisher websites, and add DOI information where available.

**********

what does this mean?). If published, this will include your full peer review and any attached files.

If you choose “no”, your identity will remain anonymous but your review may still be made public.

**Do you want your identity to be public for this peer review?**  For information about this choice, including consent withdrawal, please see our Privacy Policy

Reviewer #1: No

Reviewer #2: **Yes:**  Wisit Kaewput, MD, FRCP, FRCPSG, MFRCPT, FASN, FAcadMEd

**********

[NOTE: If reviewer comments were submitted as an attachment file, they will be attached to this email and accessible via the submission site. Please log into your account, locate the manuscript record, and check for the action link "View Attachments". If this link does not appear, there are no attachment files.]

To ensure your figures meet our technical requirements, please review our figure guidelines: https://journals.plos.org/plosone/s/figures

You may also use PLOS’s free figure tool, NAAS, to help you prepare publication quality figures: https://journals.plos.org/plosone/s/figures#loc-tools-for-figure-preparation.

NAAS will assess whether your figures meet our technical requirements by comparing each figure against our figure specifications.

# Author comment: Author response to Decision Letter 1

**DOI:** 10.1371/journal.pone.0359863.r002

13 Aug 2026

Editor’s comments:

When submitting your revision, we need you to address these additional requirements.

1. Please ensure that your manuscript meets PLOS ONE's style requirements, including those for file naming. The PLOS ONE style templates can be found at

https://journals.plos.org/plosone/s/file?id=wjVg/PLOSOne_formatting_sample_main_body.pdf and

https://journals.plos.org/plosone/s/file?id=ba62/PLOSOne_formatting_sample_title_authors_affiliations.pdf.

Response:

Thank you for this important reminder. We have carefully revised the manuscript to comply with the PLOS ONE style and formatting requirements, including the title page, author names and affiliations, section headings, references, tables, figures, figure legends, and file naming conventions. The revised manuscript and accompanying files have been prepared and named according to the PLOS ONE formatting templates and submission guidelines.

2. Thank you for stating in your Funding Statement:

“This study was supported by the Health Systems Research Institute (HSRI), Thailand (Grant No. HSRI.67-180), and Chiang Mai University. The funders had no role in study design, data collection and analysis, decision to publish, or preparation of the manuscript. “

Please provide an amended statement that declares *all* the funding or sources of support (whether external or internal to your organization) received during this study, as detailed online in our guide for authors at http://journals.plos.org/plosone/s/submit-now. Please also include the statement “There was no additional external funding received for this study.” in your updated Funding Statement.

Please include your amended Funding Statement within your cover letter. We will change the online submission form on your behalf.

Response: Thank you for this clarification. We have amended the Funding Statement to declare all sources of support received for this study, including the Health Systems Research Institute (HSRI), Thailand (Grant No. HSRI.67-180), and Chiang Mai University. We have also included the required statement, “There was no additional external funding received for this study.” The amended Funding Statement has been included in the cover letter, as requested.

3. Thank you for stating the following in the Acknowledgments Section of your manuscript:

“This study was supported by the Health Systems Research Institute (HSRI), Thailand (Grant No. HSRI.67-180), and Chiang Mai University. The funders had no role in study design, data collection and analysis, decision to publish, or preparation of the manuscript.”

We note that you have provided additional information within the Acknowledgements Section that is not currently declared in your Funding Statement. Please note that funding information should not appear in the Acknowledgments section or other areas of your manuscript. We will only publish funding information present in the Funding Statement section of the online submission form.

Please remove any funding-related text from the manuscript and let us know how you would like to update your Funding Statement. Currently, your Funding Statement reads as follows:

“This study was supported by the Health Systems Research Institute (HSRI), Thailand (Grant No. HSRI.67-180), and Chiang Mai University. The funders had no role in study design, data collection and analysis, decision to publish, or preparation of the manuscript.”

Please include your amended statements within your cover letter; we will change the online submission form on your behalf.

Response: Thank you for this important clarification. We have removed all funding-related text from the Acknowledgments section and from other sections of the manuscript. The Acknowledgments section now includes only non-financial acknowledgments.

We have included the amended Funding Statement in the cover letter, as requested, and kindly request that the Funding Statement in the online submission form be updated as follows:

“This study was supported by the Health Systems Research Institute (HSRI), Thailand (Grant No. HSRI.67-180), and Chiang Mai University. The funders had no role in study design, data collection and analysis, decision to publish, or preparation of the manuscript. There was no additional external funding received for this study.”

4. We note that your Data Availability Statement is currently as follows: [All relevant data are within the manuscript and its Supporting Information files. ]

Please confirm at this time whether or not your submission contains all raw data required to replicate the results of your study. Authors must share the “minimal data set” for their submission. PLOS defines the minimal data set to consist of the data required to replicate all study findings reported in the article, as well as related metadata and methods (https://journals.plos.org/plosone/s/data-availability#loc-minimal-data-set-definition).

For example, authors should submit the following data:

- The values behind the means, standard deviations and other measures reported;

- The values used to build graphs;

- The points extracted from images for analysis.

Authors do not need to submit their entire data set if only a portion of the data was used in the reported study.

If your submission does not contain these data, please either upload them as Supporting Information files or deposit them to a stable, public repository and provide us with the relevant URLs, DOIs, or accession numbers. For a list of recommended repositories, please see https://journals.plos.org/plosone/s/recommended-repositories.

If there are ethical or legal restrictions on sharing a de-identified data set, please explain them in detail (e.g., data contain potentially sensitive information, data are owned by a third-party organization, etc.) and who has imposed them (e.g., an ethics committee). Please also provide contact information for a data access committee, ethics committee, or other institutional body to which data requests may be sent. If data are owned by a third party, please indicate how others may request data access.

Response:

We thank the Editor for this request and confirm the following.

The quantitative data (Likert-scale responses and provider characteristics, n = 113) constitute the minimal data set for Tables 1–5 and have been de-identified and deposited in Figshare (CC BY 4.0): https://doi.org/10.6084/m9.figshare.33110660.

The qualitative data (free-text responses to three open-ended questions) come from a small, identifiable regional provider network; despite lacking direct identifiers, verbatim text carries a residual re-identification risk. Per our ethical approval, raw qualitative data are therefore not publicly deposited. Coded themes and illustrative quotations appear in the manuscript, with the full coding framework in S2 File. Anonymized data may be shared with investigators upon bona fide request and ethical approval, via the corresponding author, Prof. Kittipan Rerkasem(rerkase@gmail.com).

The Data Availability Statement has been updated accordingly:

“The de-identified quantitative minimal data set underlying the findings of this study is available at Figshare via https://doi.org/10.6084/m9.figshare.33110660. Coded qualitative thematic categories and selected illustrative quotations are reported in the manuscript, and the qualitative coding framework is provided in S2 File.

Qualitative open-ended responses were collected from a small regional provider network and are not publicly available because verbatim data may permit indirect participant identification. Requests may be directed to the corresponding author, Prof. Kittipan Rerkasem (rerkase@gmail.com), who will coordinate institutional review with the Research Institute for Health Sciences, Chiang Mai University. Access will be subject to applicable ethical approval and confidentiality requirements. Institutional contact: tel. +66 53 936148 ext. 360; fax +66 53 936059; website: https://www.rihes.cmu.ac.th/ias/ore/ .”

5. If the reviewer comments include a recommendation to cite specific previously published works, please review and evaluate these publications to determine whether they are relevant and should be cited. There is no requirement to cite these works unless the editor has indicated otherwise.

Response:

We thank the Editor for this note. No reviewer recommended specific previously published works to cite. During revision of the Discussion, we independently added one reference (Reference 22: Gale et al., Am J Nephrol 2017) to support a revised sentence on the vascular access coordinator strategy. This addition was made at the authors' discretion, not in response to a reviewer suggestion.

Additional Editor Comments (if provided):

Please revise the manuscript to clearly present the study as an exploratory, regional, provider-perception study rather than a definitive assessment of determinants of delayed AV access creation. Please address the following key points:

1. Revise the abstract, results, discussion, and conclusion to avoid overgeneralization. The findings should be framed as perceived barriers and facilitators among providers in Upper Northern Thailand, not as nationally representative or causal findings.

Response:

We thank the Editor for this comment. We revised the Abstract, Results, Discussion, and Conclusion to consistently frame findings as provider-perceived, exploratory, and regional rather than nationally representative or causal. Key changes:

Abstract — Aim reframed as: “This exploratory regional study compared the perspectives of nephrologists, vascular surgeons, and dialysis nurses in Upper Northern Thailand and identified supportive strategies that providers considered relevant…” (p. 3, lines 58–61). Conclusions rewritten to: “Providers in this regional study identified shared priorities for improving timely predialysis AV access preparation, while differing in the meanings and considerations they associated with those priorities. These context-specific, hypothesis-generating findings may inform locally adapted service improvement…” (p. 4, lines 84–88).

Results — Added disclaimers not in the original, e.g., Table 3 note: “Qualitative excerpts are illustrative and provided for explanatory purposes; they are not intended to indicate prevalence, dominance, or relative importance” (p. 17, lines 385–386), repeated under Tables 4 and 5.

Discussion (p. 20–21) — Opening now identifies the study as exploratory and regional, describing “findings represent provider-reported, hypothesis-generating insights” (lines 449–450). Explicit caution against causal interpretation added, e.g.: “The present study did not directly examine why rating differed across professional groups… and warrant further investigation using objective clinical and service data” (lines 482–486); “Because patients and families were not directly surveyed, the patient-level findings represent providers' interpretations…rather than direct patient-reported evidence” (lines 472–474).

Conclusion (p. 28, lines 668–674) — Fully rewritten to: “In this exploratory regional provider-perception study, healthcare providers in Upper Northern Thailand identified…These context-specific, hypothesis-generating findings may inform locally adapted service improvement and the design of future evaluations.”

2. Clarify the sampling strategy and its limitations, including recruitment through an academic meeting/professional networks, possible selection bias, small numbers of nephrologists and vascular surgeons, and the predominance of dialysis nurses.

Response:

We thank the Editor for highlighting this issue. We clarified the sampling strategy in the Methods and expanded the Limitations section.

Methods, Participants, setting, and recruitment (p. 9–10, lines 210–215): “Recruitment took place between February and April 2025 using a pragmatic, opportunity-based approach. Participants were recruited primarily through a regional academic meeting on vascular access held at the Faculty of Medicine, Chiang Mai University, supplemented by targeted invitations distributed through established professional networks…”

Limitations (p. 27, lines 636–646) — on the predominance of dialysis nurses and small nephrologist/surgeon numbers: “…the professional subgroups were uneven, with dialysis nurses comprising most participants and relatively small numbers of nephrologists and vascular surgeons… The nursing findings therefore should not be considered representative of all nursing roles in the predialysis pathway.”

Limitations (p. 27, lines 647–649) — on selection bias: “Third, pragmatic recruitment through a regional vascular access meeting and professional networks may have introduced selection bias by preferentially including providers with greater interest or engagement in vascular access care.”

3. Address the concern that dialysis nurses may provide mainly retrospective views of predialysis care. Please clarify whether any nurses had active CKD stage 4–5 or predialysis access-planning roles. If CKD/predialysis nurses were not included, this should be acknowledged as a limitation.

Response:

We thank the Editor for this important point. We confirm that dedicated CKD/predialysis (OPD nephrology) nurses were not sampled as a separate subgroup, and this is acknowledged explicitly:

Methods, Participants, setting, and recruitment (p. 9–10, lines 199–207): “Eligible participants were healthcare providers directly involved in chronic kidney disease (CKD) management, vascular access planning and coordination, renal replacement therapy education, and hemodialysis care… In some regional hospital settings, dialysis nurses participate in predialysis renal replacement therapy counseling, including education for patients and families regarding dialysis modalities…”

Discussion, Role-related variation in perceived obstacles (p. 22, lines 502–508): “In some hospitals in Upper Northern Thailand, dialysis nurses also participate in predialysis renal replacement therapy counseling, although the extent of this involvement was not measured for individual respondents. The nursing findings therefore reflect dialysis nurses' perspective arising from differing combinations of predialysis counseling and experience with patients transitioning to hemodialysis, rather than the full range of nursing roles across the predialysis pathway.”

Limitations (p. 27, lines 640–646): “In some regional hospitals, dialysis nurses participate in predialysis counselling, whereas in others setting their perspectives may arise primarily from caring for patients transitioning to hemodialysis. The extent of individual nurses' involvement in predialysis involvement was not measured, and dedicated CKD or predialysis nurses were not sampled separately. The nursing findings therefore should not be considered representative of all nursing roles in the predialysis pathway.”

For context (not a measured finding, since individual-level involvement was not collected): the “regional hospital settings” referred to above are predominantly government/provincial hospitals, where 62% (48/78) of nurse participants practiced (Table 1) — settings where dialysis nurses commonly assist with predialysis counselling as part of routine practice. This remains appropriately reported as a limitation rather than a quantified finding.

4. Clarify that patient-level barriers reflect provider perceptions only. The absence of direct patient perspectives and objective clinical/system data, such as catheter use at initiation, AVF/AVG creation rates, referral-to-surgery time, surgical wait time, and reimbursement delays, should be explicitly discussed.

Response:

We thank the Editor for this important point. We agree, and have made this distinction explicit at four points in the manuscript:

Results, Integrated barriers section (p. 16, lines 356–358): “These findings represent providers' perceptions rather than direct patient-reported or audited clinical and service indicators.”

Discussion, Shared and varying perceptions section (p. 21, lines 472–474): “Because patients and families were not directly surveyed, the patient-level findings represent providers' interpretations of patient readiness and decision-making rather than direct patient-reported evidence.”

Table 1 note (p. 15, lines 337–338): “These provider-estimated proportions of predialysis AV access were self-reported by providers and are presented as contextual practice estimates, not audited institutional rates.”

Limitations, closing paragraph (p. 28, lines 654–657) — explicitly naming each objective indicator raised by the Editor: “…the study did not include objective measures of catheter use at dialysis initiation, audited AVF or AVG creation rates, referral completion, referral-to-procedure intervals, failed referrals, procedural waiting time, reimbursement delays, or patient-level reasons for noncompletion.”

5. Strengthen the description of questionnaire development and validation, including item generation, expert review, pilot testing, item revision, and interpretation of Cronbach’s alpha.

Response:

We thank the Editor for this recommendation. We expanded the “Questionnaire development” subsection (now split into “Item generation” and “Content validation and pilot testing”) in the main manuscript, and added a fuller account in S1 File.

Item generation (p. 8–9, lines 176–179): clarified how conceptual categories from initial provider interviews were converted into items: “Conceptual categories were then mapped to candidate questionnaire items by the research team, with items retained when they reflected recurring provider-identified barriers or facilitators and could be rated across one or more professional groups”.

Content validation and pilot testing (p. 9, lines 187–198): specified expert-review criteria, pilot sample size, and evaluation domains: “…reviewed by three subject-matter experts…to assess content validity, clarity, domain coverage, and relevance across the three professional groups. Revisions were made to improve item wording…Pilot testing was then conducted with 6 providers to evaluate feasibility, comprehension, completion time, and the clarity of instructions and response options.”

Cronbach's alpha interpretation (p. 12, lines 261–266): “Given its modest internal consistency, the patient-level barrier domain was interpreted descriptively and was not treated as a validated unidimensional patient-readiness construct.”

S1 File now documents the full process, including the semi-structured elicitation guide administered separately to each professional group, and states explicitly that “Cronbach's alpha was used to assess internal consistency of predefined item domains; however, internal consistency alone was not considered evidence of full psychometric validation.”

Together, these revisions provide a transparent account of item generation, expert content review, pilot testing, item refinement, and appropriately cautious interpretation of internal consistency.

6. Clarify the statistical analysis, including missing data, p-values, multiple comparisons, use of domain-level Likert scores, and the absence of a formal sample-size calculation. Cross-professional comparisons should be presented as exploratory.

Response

We thank the Editor for this recommendation. We clarified the statistical analysis in the Methods section as follows.

Sample-size calculation (p. 10, lines 217–220): “A formal sample size calculation was not undertaken because the study was exploratory and was not designed to test a predetermined effect size. The final sample comprised 78 dialysis nurses, 19 vascular surgeons, and 16 nephrologists and was used for descriptive cross professional comparisons and identification of recurring concepts in the open-ended responses.”

Domain-level Likert scores (p. 11, lines 251–254): “Participant-level domain summaries were calculated by averaging responses to conceptually related items within each predefined domain; medians and IQRs of these summaries were reported descriptively. Domain summaries were not used to select items for the joint displays or to derived the principal integrated findings.”

Missing data, p-values, and multiple comparisons (p. 11, lines 255–260): “There were no missing responses for core barrier, profession-specific obstacle, or supportive-factor items; non-response occurred only for some open-ended questions. Exact Kruskal–Wallis p-values are reported in S3 File. Given the exploratory analyses, multiple item comparisons, and small professional subgroups, p-values were not adjusted for multiplicity and were interpreted descriptively rather than as confirmatory evidence of between-group differences.”

Consistent with our response to Comment 6, cross-professional comparisons throughout the Abstract, Results, Discussion, and Conclusion are now presented as exploratory and descriptive, not confirmatory.

7. Clarify the mixed-methods component. Please state the nature and depth of the qualitative data, the number of open-ended responses, coding procedures, and how qualitative and quantitative findings were integrated.

Response

We thank the Editor for this recommendation. We clarified the qualitative component and mixed-methods integration process as follows.

Data collection procedures (p. 10–11, lines 237–241): “The qualitative component comprised brief written responses to three open-ended survey questions rather than in-depth interviews or focus groups. Overall, 97 of 113 participants provided at least one open-ended response; 94 responded to the question on key facilitators, 92 identified an urgent obstacle, and 68 provided suggestions for improvement.”

Qualitative analysis (p. 12, lines 274–286): “Open-ended responses were analyzed using a combined deductive–inductive approach. Given the brief written format, the analysis was intended to identify recurring concepts and provide contextual detail for the quantitative response patterns rather than to develop an in-depth interpretive theory…Coding decisions, theme definitions, and representative excerpts were reviewed iteratively by the research team. The complete coding framework and analytic process are provided in S2 File.”

Mixed-methods integration (p. 13, lines 302–306): “The joint displays are illustrative rather than exhaustive and were not intended to rank barriers or supportive factors by prevalence or importance. Qualitative excerpts were used to show how participants described the selected items and were not treated as frequency estimates.”

8. Ensure transparent reporting of selected results. If Tables 3–5 present selected items, please provide complete item-level results in the supplementary materials and describe the joint displays as illustrative.

Response

We thank the Editor for this recommendation. Tables 3–5 are now explicitly described as illustrative, and complete item-level results are provided in S3 File.

Main manuscript, Mixed-methods integration and selection of joint display items (p. 13, lines 302–306): “The joint displays are illustrative rather than exhaustive and were not intended to rank barriers or supportive factors by prevalence or importance. Qualitative excerpts were used to show how participants described the selected items and were not treated as frequency estimates. Complete item-level results, p-values, and the rationale for item selection are provided in S3 File.”

Results section — each table is individually cross-referenced to its full item-level dataset:

• Barriers (Table 3), p. 16, lines 357–358: “Complete quantitative results are provided in S3 File part A.”

• Role-specific obstacles (Table 4), p. 17, lines 394–395: “Full quantitative results for all role-specific obstacle items are provided in S3 File part B.”

• Supportive factors (Table 5), p. 19, lines 427–428: “Complete quantitative results for all supportive-factor items are provided in S3 File part C.”

S3 File was restructured into three matching parts (S3-A, S3-B, S3-C), each providing: (i) full item-level medians and IQRs by professional role for every questionnaire item, not only the items shown in Tables 3–5; (ii) exact Kruskal–Wallis p-values; and (iii) an explicit statement of the predefined selection criteria used for each table. The file states directly: “Items were selected to illustrate analytically relevant patterns for mixed-methods integration, rather than to exhaustively represent or rank all measured questionnaire items.”

Together, these revisions ensure that the selective, illustrative nature of Tables 3–5 is stated transparently in the manuscript, and that readers can access the complete underlying item-level results in S3 File.

9. Revise Figure 1 to improve conceptual consistency. In particular, “procedural wait time” appears to be a system-level barrier rather than a patient/family readiness factor.

Response

We thank the Editor for identifying this inconsistency. We agree that procedural wait time is a system-level capacity barrier, not a patient/family readiness factor, and revised Figure 1 accordingly.

Original submission, Figure 1: two boxes were both labeled “Patient & Family Readiness” — one listing “Limited understanding/acceptance; Late presentation,” and a duplicated box listing only “Procedural wait time.”

Revised Figure 1: the duplicated box was removed, and “procedural wait time” relocated into “System Capacity & Access” (alongside OR/service availability, staffing, and vascular assessment resources), consistent with its classification in Table 3 (p. 17).

Figure 1 caption (p. 25, lines 579–586) was revised: “The figure integrates provider-reported quantitative and qualitative findings across the predialysis AV access care pathway. Providers shared several priorities but emphasized different aspects according to professional role. The provider-endorsed strategies shown are candidate components for locally adapted service improvement and future evaluation. The figure is illustrative and does not represent a causal pathway or observed service implementation.”

This aligns the figure's visual classification with the quantitative domain assignment in Table 3, and improves conceptual consistency.

10. Revise Table 1 to improve workload reporting across professional groups. If nephrologists’ active dialysis patient workload was not collected, please state this.

Response

We thank the Editor for this clarification. We revised the Results text and Table 1 note to state explicitly that nephrologists' active maintenance-hemodialysis workload was not collected.

Results, Participant characteristics (p. 14, lines 322–327): “Clinical workload was summarized using role-specific measures collected in the questionnaire. Nurses reported a median of 120 dialysis patients under their care per month (IQR 64–275), surgeons reported a median of nine AV access procedures per month (IQR 5–12), and nephrologists managed a median of 150 patients with CKD stages 4–5 per month (IQR 58–200). Maintenance-hemodialysis workload was not collected for nephrologists.”

Table 1 note (p. 15, lines 335–337): “Workload measures were role-specific. Nephrologists' active maintenance hemodialysis patient workload was not collected.”

11. Please also shorten and focus the Discussion, reduce repetitive conceptual language, and distinguish clearly between observed findings, interpretation, and hypotheses for future service improvement.

Response

We thank the Editor for this recommendation. We shortened the Discussion, removed repetitive conceptual language, and restructured it to distinguish observed findings from interpretation and forward-looking hypotheses.

Length reduced from ~2,500 words (original) to ~1,900 words (revised).

Repetitive conceptual language removed: “operational misalignment” ecurred 7 times as a central organizing concept) was removed entirely and replaced with more direct language; “role-specific” reduced from 20 to 12 occurrences.

Clearer structure (p. 20–26), reflected in revised subheadings:

• “Shared and varying perceptions of patient, system, and clinical barriers” (p. 20, line 452) and “Role-related variation in perceived obstacles” (p. 22, line 493) present the observed quantitative and qualitative findings.

• “Shared endorsement of supportive strategies and priorities for evaluation” (p. 23, line 535) interprets what providers converged on.

• “Implications for service improvement and future evaluation” (p. 25, line 590) is now placed in its own section after the findings are presented, clearly signaling to the reader which content is exploratory and forward-looking.

The Discussion opening (p. 20, lines 449–450) now explicitly labels the contribution: “These findings represent provider-reported, hypothesis-generating insights intended to inform future service evaluation,” signaling from the outset that subsequent interpretive content should not be read as confirmed or causal.

Reviewers’ comments:

Reviewer #1:

The idea of conducting this study is practically reasonable. However, after reviewing the article, I have the following comments.

1. Methodological Misalignment in Sample Selection: A major limitation of this study is the heavy reliance on dialysis nurses, who constitute 69% of the total sample, to evaluate predialysis vascular access planning. Dialysis nurses primarily care for patients who have already initiated hemodialysis, meaning their insights into predialysis barriers are retrospective and subject to recall bias. The study notably omits the perspectives of Chronic Kidney Disease (CKD) or predialysis nurses, who are actively responsible for patient education, tracking readiness, and coordinating care during CKD stages 4 and 5. Consequently, the dominant nursing voice in this paper reflects post-dialysis observations rather than active, frontline management of the predialysis pathway, potentially skewing the validity of the identified workflow constraints.

Response:

We thank the Reviewer for this important observation. This overlaps substantially with Editor Comments 7 and 8on nurse predominance, selection bias, and the absence of dedicated CKD/predialysis nurses. We summarize the corresponding revisions here.

Methods, Participants, setting, and recruitment (p. 9–10, lines 200–222): the final distribution (78 dialysis nurses, 19 vascular surgeons, 16 nephrologists) is reported (lines 218–219), with eligibility criteria described as covering “healthcare providers directly involved in chronic kidney disease (CKD) management, vascular access planning and coordination, renal replacement therapy education, and hemodialysis care.” (lines 200–202)

Limitations (p. 27, lines 636–653) now explicitly acknowledges:

• Nurse predominance and its effect on cross-professional balance: “…the professional subgroups were uneven, with dialysis nurses comprising most participants and relatively small numbers of nephrologists and vascular surgeons… The nursing findings therefore should not be considered representative of all nursing roles in the predialysis pathway.” (lines 636–646)

• That dedicated CKD/predialysis nurses were not sampled separately, and individual nurses' extent of predialysis involvement was not measured (lines 640–646).

• Selection bias: “…pragmatic recruitment through a regional vascular access meeting and professional networks may have introduced selection bias by preferentially including providers with greater interest or engagement in vascular access care” (lines 647–649).

Limitations, self-report/recall bias (p. 26–27, lines 625–628) — directly addressing the Reviewer's concern about retrospective, recall-based reporting: “…the study examined provider-reported perceptions rather than direct patient or family experiences, observed care processes, or objective clinical outcomes. Self-reported responses may have been affected by recall, reporting, or social desirability bias.”

Discussion, Role-related variation in perceived obstacles (p. 22, lines 502–508) further clarifies that nursing findings reflect a mix of predialysis counselling involvement and post-transition experience, “rather than the full range of nursing roles across the predialysis pathway.”

These revisions transparently acknowledge that the nursing perspective may include a retrospective component, that dedicated CKD/predialysis nurses were not sampled, and that findings should not be generalized beyond this sample.

2. Psychometric Instability Linked to Proxy Reporting: The suboptimal internal consistency of the patient-level domain (Cronbach's alpha = 0.67) serves as direct statistical evidence of a fundamental design flaw: attempting to measure patient-level constructs through proxy provider perspectives. Because healthcare providers—particularly dialysis nurses who only manage patients retrospectively—must speculate on the behavioral and educational barriers of the predialysis phase, their ratings might be suboptimal. This mismatch causes high variance and poor item-to-item correlation within the subscale. Had the authors directly surveyed or interviewed the patients themselves, the items mapping onto patient readiness would likely have demonstrated significantly higher psychometric reliability and conceptual cohesion.

Response:

We thank the reviewer for this comment. We agree the patient-level domain showed only modest internal consistency and should not be interpreted as a validated patient-readiness scale or direct patient-reported evidence. We revised the manuscript to clarify these items describe heterogeneous provider-perceived patient-level barriers, not a unidimensional patient construct.

Quantitative measures and analysis (p.12, lines 261–266): “Internal consistency was assessed using Cronbach's alpha. For core barrier items, alpha was 0.67 for the patient-level domain (5 items), 0.71 for the system-level domain (4 items), and 0.82 for the overall barrier scale (10 items). Given its modest internal consistency, the patient-level barrier domain was interpreted descriptively and was not treated as a validated unidimensional patient-readiness construct.”

Results (p.16, lines 356–357): “These findings represent providers' perceptions rather than direct patient-reported or audited clinical and service indicators.”

Strengths and limitations (p.26, lines 625–632): “...the study examined provider-reported perceptions rather than direct patient or family experiences, observed care processes, or objective clinical outcomes. Self-reported responses may have been affected by recall, reporting, or social desirability bias. Patient-level items should therefore not be interpreted as patient-reported determinants of readiness or decision-making. In addition, the modest internal consistency of the patient-level domain supports treating these items as heterogeneous provider-perceived concerns rather than as a validated patient-readiness construct.”

We agree direct patient/family perspectives would add valuable evidence and have noted this as a limitation. The study's purpose, however, was to examine provider-reported perceptions across roles, not to validate a psychometric patient-readiness instrument.

3. Suboptimal Definition of the Target Patient Phenomenon: A fundamental methodological weakness of this study is its broad, undifferentiated focus on all patients initiating dialysis without permanent access. A substantial portion of advanced CKD patients are clinically or anatomically unsuitable for AVF creation due to vascular exhaustion or severe comorbidities. To effectively isolate system-level and behavioral barriers to timely planning, the study should have utilized a targeted case-selection strategy. Specifically, it should have focused on the subset of patients who were clinically viable candidates for an AVF but still initiated dialysis via a catheter before subsequently undergoing successful AVF creation. Interviewing the specific providers managing this precise “delayed-but-eligible” cohort would have yielded highly actionable insights into care-pathway bottlenecks, rather than capturing generalized perceptions of a heterogeneous patient pool.

Response:

We thank the reviewer for this thoughtful suggestion. We agree that a targeted study of a clinically eligible “delayed-but-eligible” cohort would be valuable, and that not all advanced CKD patients are suitable candidates for AVF/AVG creation due to vascular anatomy, comorbidity, or other clinical factors.

The present study, however, was not designed as a patient-level cohort study or chart review to determine clinical eligibility, catheter initiation, or audited AVF/AVG outcomes. It was designed as an exploratory regional provider-perception study comparing how nephrologists, vascular surgeons, and dialysis nurses perceived barriers and supportive factors across the predialysis AV access pathway. This framing is maintained throughout (Abstract, line 58; Discussion, line 439; Conclusion, line 668).

Results (p.16, lines 356–357): “…These findings represent providers' perceptions rather than direct patient-reported or audited clinical and service indicators.”

Strengths and limitations (p.28, lines 659–663): “...the study also did not identify a clinically eligible 'delayed-but-eligible' patient cohort or evaluate the feasibility, implementation, or effectiveness of the endorsed strategies. Accordingly, the findings identify provider-perceived barriers and priorities for future evaluation rather than determinants of actual delayed access creation.”

We also retained clinical suitability as a provider-perceived barrier: the questionnaire included items on comorbidities affecting suitability (Table 3) and technical complexity of AV access procedures (Table 4), and the qualitative findings include “Context-specific Technical/Clinical Suitability Considerations” as a theme (Table 2). While the study does not isolate a delayed-but-eligible cohort, it acknowledges clinical suitability as part of providers' interpretation of delayed AV access planning and creation.

4. Inconsistent Workload Metrics and Data absence in Table 1: In Table 1, the authors fail to report the number of active dialysis patients managed per month by participating nephrologists, leaving a notable data gap in the descriptive statistics. While dialysis nurses' workloads are quantified by “dialysis patients/month”, nephrologists are only evaluated on their predialysis CKD 4–5 volume. In clinical practice, particularly in Thailand, nephrologists actively manage and round on maintenance hemodialysis patients.

Response:

We thank the reviewer for this observation. We agree nephrologists in Thailand often manage and round on maintenance hemodialysis patients. However, the questionnaire collected role-specific workload measures rather than a common metric across groups: dialysis nurses reported dialysis patients under their care per month, vascular surgeons reported AV access procedures per month, and nephrologists reported CKD stage 4–5 patients per month. Nephrologists' active maintenance hemodialysis workload was not collected and therefore could not be reported.

Results (p.14, lines 322–327): “Clinical workload was summarized using role-specific measures collected in the questionnaire. Nurses reported a median of 120 dialysis patients under their care per month (IQR 64–275), surgeons reported a median of nine AV access procedures per month (IQR 5–12), and nephrologists managed a median of 150 patients with CKD stages 4–5 per month (IQR 58–200). Maintenance-hemodialysis workload was not collected for nephrologists.”

Table 1 note (p.15, lines 336–337): “Workload measures were role-specific. Nephrologists' active maintenance hemodialysis patient workload was not collected.”

These revisions clarify that the Table 1 workload variables were role-specific descriptive measures, and that nephrologists' maintenance hemodialysis workload was not assessed in the questionnaire.

5. Conceptual Inconsistency and Poor Visual Flow in Figure 1: The conceptual model presented in Figure 1 contains significant logical contradictions and fails to establish a well-connected flow linking specific barriers to the stages of the predialysis care pathway. Notably, “Procedural wait time” is placed under the domain of “Patient & family readiness”. This probably mischaracterizes a structural, system-level capacity barrier as a patient-level factor and directly contradicts the authors' own classification in Table 3, where it is correctly labeled as a system-level barrier. Furthermore, the duplicated labeling of “Patient & family readiness” boxes on the left side of the diagram reflects poor visual creation.

Response:

We thank the reviewer for this careful assessment. We agree with both concerns: the previous figure mischaracterized “procedural wait time” as a patient-level factor despite classifying it as system-level in Table 3, and it contained a duplicated Patient and Family Readiness box that impaired visual flow.

We revised Figure 1 to address both issues. “Procedural wait time” is now placed under System Capacity and Access, consistent with Table 3, and the duplicated box was removed. We also reorganized the figure to show a clearer flow across sequential stages of the predialysis AV access pathway — CKD recognition, planning (RRT education and modality counseling), referral, preparation (surgical evaluation), procedure (AVF/AVG creation and follow-up), and hemodialysis initiation with planned AV access — with provider-perceived barriers aligned above this pathway and provider-endorsed strategies shown as candidate components below it, rather than embedded within the barrier domains.

The caption was revised to clarify the model is illustrative rather than a definitive causal pathway:

“The figure integrates provider-reported quantitative and qualitative findings across the predialysis AV access care pathway. Providers shared several priorities but emphasized different aspects according to professional role. The provider-endorsed strategies shown are candidate components for locally adapted service improvement and future evaluation. The figure is illustrative and does not represent a causal pathway or observed service implementation.” (p.25, lines 581–586)

Reviewer #2:

This manuscript addresses an important and clinically relevant issue: delayed predialysis arteriovenous access planning and creation before hemodialysis initiation in Thailand. The topic is worthwhile, and the attempt to compare perspectives among nephrologists, vascular surgeons, and dialysis nurses is potentially valuable. Understanding multidisciplinary barriers to timely AV access preparation is relevant for improving predialysis care pathways, reducing catheter dependence, and informing service planning.

However, the manuscript requires major revision before it can be considered further. The study has potential, but several important methodological, analytic, and reporting issues need to be addressed. In particular, the conclusions should be made more cautious and better aligned with the exploratory nature of the data.

1. The first major concern relates to the sampling strategy and representativeness. The study is described as a cross-sectional convergent mixed-methods study, but recruitment was pragmatic and opportunity-based, relying primarily on a regional vascular access academic meeting and professional networks. This approach is understandable for an exploratory study, but it introduces a substantial risk of selection bias. Providers attending such a meeting or reachable through professional networks may already be more engaged with vascular access planning than the broader provider population. The authors should discuss this limitation more explicitly and avoid implying that the findings represent all providers in Northern Thailand or Thailand as a whole.

Response:

We thank the reviewer for this comment. We agree pragmatic, opportunity-based recruitment may introduce selection bias and that the findings are not nationally representative. We revised the manuscript to clarify recruitment and frame the study as exploratory and regional to Upper Northern Thailand.

Participants, setting, and recruitment (p. 10, lines 210–215): “Recruitment took place between February and April 2025 using a pragmatic, opportunity-based approach. Participants were recruited primarily through a regional academic meeting on vascular access held at the Faculty of Medicine, Chiang Mai University, supplemented by targeted invitations distributed through established professional networks to nephrologists, vascular surgeons, and dialysis nurses practicing in the region.”

Abstract (p.3, lines 58–61): “This exploratory regional study compared the perspectives of nephrologists, vascular surgeons, and dialysis nurses in Upper Northern Thailand and identified supportive strategies that providers considered relevant to improving timely AV access preparation.”

Strengths and limitations (p.27, lines 647–653): “...pragmatic recruitment through a regional vascular access meeting and professional networks may have introduced selection bias by preferentially including providers with greater interest or engagement in vascular access care. Participants were drawn from Upper Northern Thailand, where service organization, workforce capacity, referral arrangements, and reimbursement processes may differ from those in other regions. The findings are therefore context-specific and are not representative of all providers in Thailand.”

Also (p.26–27, lines 625–628): “...the study examined provider-reported perceptions rather than direct patient or family experiences, observed care processes, or objective clinical outcomes. Self-reported responses may have been affected by recall, reporting, or social desirability bias.”

These revisions clarify that recruitment was pragmatic and regionally bound, the sample may over-represent providers with greater engagement in vascular access care, and findings should be read as context-specific to Upper Northern Thailand rather than representative of Thailand as a whole.

2. The sample size and imbalance across professional groups also require more careful handling. Although the total sample included 113 participants, the subgroup sizes were uneven, with 78 nurses, 19 vascular surgeons, and only 16 nephrologists. These small numbers limit the reliability of cross-professional comparisons. The manuscript repeatedly emphasizes role-specific differences, workflow misalignment, and operational differences across professional groups. These observations may be useful, but they should be framed as exploratory and hypothesis-generating rather than definitive. The authors should revise the Results, Discussion, and Conclusion to avoid overinterpreting small subgroup differences.

Response:

We agree that uneven subgroup sizes limit cross-professional comparisons. We revised the Results, Discussion, and Conclusion to present role-specific differences as exploratory and hypothesis-generating rather than definitive, and to avoid overinterpreting small subgroup differences.

Quantitative measures and analysis (p.11, lines 248–260): “Because the questionnaire was newly developed and the Likert responses were ordinal, the primary quantitative interpretation was conducted at the item level... Given the exploratory analyses, multiple item comparisons, and small professional subgroups, p-values were not adjusted for multiplicity and were interpreted descriptively rather than as confirmatory evidence of between-group differences.”

Role-related variation in perceived obstacles (p.22, lines 496–498): “These are descriptive because the items differed across professions, subgroup sizes were uneven, and the three sets of role-specific items were not directly compared statistically.”

Discussion opening (p.20, lines 449–450): “These findings represent provider-reported, hypothesis-generating insights intended to inform future service evaluation.”

Also (p. 23, lines 528–533): “The patterns do not necessarily indicate disagreement across professions; rather, they show how shared priorities may be understood through different professional lenses. This interpretation generates testable questions for future studies of communication, referral processes, task allocation, and clinical outcomes across the AV access pathway.”

Conclusion (p.28, lines 672–674): “These context-specific, hypothesis-generating findings may inform locally adapted service improvement and the design of future evaluations.”

Together, these revisions clarify that role-specific patterns should be read as exploratory, hypothesis-generating insights rather than definitive comparative conclusions given the uneven subgroup sizes.

3. The absence of a formal sample-size calculation should also be addressed more clearly. The authors state that the study was exploratory and not intended to test a predetermined effect size. This is acceptable, but the manuscript should then consistently present the quantitative findings as descriptive. The authors should avoid language suggesting strong comparative inference unless supported by appropriate statistical evidence.

Response:

We thank the reviewer for this clarification. We agree that, absent a formal sample-size calculation, the quantitative findings should be interpreted as descriptive and exploratory rather than definitive comparative evidence.

Participants, setting, and recruitment (p.10, lines 217–218): “A formal sample size calculation was not undertaken because the study was exploratory and was not designed to test a predetermined effect size.”

Quantitative measures and analysis (p.11, lines 257–260): “Exact Kruskal–Wallis p-values are reported in S3 File. Given the exploratory analyses, multiple item comparisons, and small professional subgroups, p-values were not adjusted for multiplicity and were interpreted descriptively rather than as confirmatory evidence of between-group differences.”

We also revised the Abstract, Results, Discussion, and Conclusion to consistently frame cross-professional findings as provider-reported, exploratory, and hypothesis-generating, avoiding language implying definitive between-group differences or causal inference.

For consistency, S3 File (Full List of Quantitative Item and Descriptive Statistics) now states, for both the barrier-item and supportive-factor tables: “P-values were not adjusted for multiple comparisons because the quantitative analyses were exploratory and intended to describe response patterns for mixed-methods integration rather than to test confirmatory hypotheses.” This ensures the exploratory, non-confirmatory interpretation of p-values is stated consistently across the main manuscript and all supplementary item-level results.

4. The mixed-methods component needs clearer justification and more cautious interpretation. The manuscript presents the study as a convergent mixed-methods design, but the qualitative component appears to be based on brief open-ended survey responses rather than in-depth interviews or focus groups. Open-ended survey responses can provide useful contextual information, but they usually provide limited depth. Therefore, claims about underlying mechanisms, workflow dynamics, or operational misalignment should be softened. The authors should clarify the depth and nature of the qualitative data, report how many participants provided open-ended responses, and explain how coding reliability or analytic credibility was ensured.

Response:

We thank the reviewer for this important comment. We agree the qualitative component was based on brief written open-ended survey responses rather than in-depth interviews or focus groups, and interpretation should be cautious. We revised the manuscript to clarify the nature and depth of the qualitative data and soften claims regarding mechanisms and workflow dynamics.

Data collection procedures (p.11, lines 237–241): “The qualitative component comprised brief written responses to three open-ended survey questions rather than in-depth interviews or focus groups. Overall, 97 of 113 participants provided at least one open-ended response; 94 responded to the question on key facilitators, 92 identified an urgent obstacle, and 68 provided suggestions for improvement.”

Qualitative analysis (p.12, lines 275–286): “Given the brief written format, the analysis was intended to identify recurring concepts and provide contextual detail for the quantitative response patterns rather than to develop an in-depth interpretive theory………Coding decisions, theme definitions, and representative excerpts were reviewed iteratively by the research team.”

A dedicated reflexivity statement further supports analytic credibility (p.13, lines 307–310): “A reflexivity and analytic rigor statement detailing researcher positioning, analytic decision-making, and strategies to enhance credibility and transparency is provided in S4 File.”

Mixed-methods integration and selection of joint display items (p.13, lines 302–305): “The joint displays are illustrative rather than exhaustive and were not intended to rank barriers or supportive factors by prevalence or importance. Qualitative excerpts were used to show how participants described the selected items and were not treated as frequency estimates.” This is reinforced in the notes to Tables 3, 4, and 5: “Qualitative excerpts are illustrative and provided for explanatory purposes; they are not intended to indicate prevalence, dominance, or relative importance.”

We also reviewed the Results, Discussion, and Conclusion to ensure that terms such as workflow dynamics and operational misalignment are presented as provider-perceived and hypothesis-generating, rather than as definitive mechanistic claims.

5. The questionnaire development and validation process should be described in more detail. The manuscript states that exploratory interviews informed item development, followed by expert review and pilot testing. However, important details are missing, including how interview findings were translated into survey items, how content validity was assessed, how pilot testing was performed, whether items were revised after pilot testing, and whether the instrument was assessed for clarity across all three professional groups. Cronbach’s alpha values are helpful, but internal consistency alone does not establish validity, particularly for a newly developed instrument. The authors should strengthen this section and consider moving the full questionnaire and validation process into a clearly described supplementary file.

Response:

We thank the reviewer for this recommendation. We agree the questionnaire development and content validation process required clearer description, and that Cronbach's alpha alone does not establish validity for a newly developed instrument.

Methods (p.8, lines 176–178) clarifies how interview findings were translated into items: “Conceptual categories were then mapped to candidate questionnaire items by the research team, with items retained when they reflected recurring provider-identified barriers or facilitators and could be rated across one or more professional groups.”

Content validation and pilot testing (p.9, lines 188–198): “The draft questionnaire was reviewed by three subject-matter experts in clinical nephrology, vascular access surgery, and mixed-methods research to assess content validity, clarity, domain coverage, and relevance across the three professional groups. Revisions were made to improve item wording, reduce ambiguity, and ensure that role-specific items were appropriate for dialysis nurses, vascular surgeons, and nephrologists. Pilot testing was then conducted with 6 providers to evaluate feasibility, comprehension, completion time, and the clarity of instructions and response options. Feedback was used to refine item wording and survey format before final administration. The questionnaire was developed for descriptive use in this exploratory provider-perception study and was not intended as a fully validated psychometric instrument.”

To address cross-professional clarity assessment, S1 File was expanded: “Pilot testing was then conducted with 6 providers (2 dialysis nurses, 2 vascular surgeons, and 2 nephrologists) to evaluate feasibility, comprehension, completion time, clarity of instructions, and clarity of response options.”

Quantitative measures and analysis (p.12, lines 261–266) clarifies Cronbach's alpha assessed internal consistency of predefined domains, not full psychometric validation; the patient-level barrier domain was interpreted descriptively given its modest internal consistency and was not treated as a validated unidimensional construct.

S1 File was also expanded to include the full questionnaire development, content validation, and pilot testing process, with the title updated to: “S1 File. Questionnaire development, content validation, pilot testing, and full questionnaire.”

These revisions provide a clearer account of item generation, expert review, pilot testing, item refinement, cross professional clarity assessment, and the appropriate interpretation of Cronbach's alpha.

6. The statistical analysis also requires clarification. Likert-scale responses were summarized using medians and interquartile ranges and compared using Kruskal-Wallis tests, which is reasonable for exploratory analysis. However, the manuscript should more clearly report p-values, missing-data handling, and whether any adjustment for multiple comparisons was considered. If no adjustment was applied, this should be stated and justified. The authors should also consider reporting effect sizes or emphasizing descriptive patterns rather than statistical significance, given the small subgroup sizes.

Response:

We thank the reviewer for this clarification. We revised the Quantitative measures and analysis section to clarify p-value reporting, missing-data handling, multiple-comparison considerations, and the exploratory interpretation of statistical comparisons.

Quantitative measures and analysis (p.11, lines 255–260): “There were no missing responses for core barrier, profession-specific obstacle, or supportive-factor items; non-response occurred only for some open-ended questions. Exact Kruskal–Wallis p-values are reported in S3 File. Given the exploratory analyses, multiple item comparisons, and small professional subgroups, p-values were not adjusted for multiplicity and were interpreted descriptively rather than as confirmatory evidence of between-group differences.”

For consistency, the S3-A and S3-C table footnotes state: “P-values were not adjusted for multiple comparisons because the quantitative analyses were exploratory and intended to describe response patterns for mixed-methods integration rather than to test confirmatory hypotheses.”

The Discussion (p.22, lines 496–498) similarly notes role-specific comparisons were not tested statistically, given small and uneven subgroup sizes: “These are descriptive because the items differed across professions, subgroup sizes were uneven, and the three sets of role-specific items were not directly compared statistically.”

Regarding effect sizes, we considered this suggestion carefully. Given the exploratory nature of the quantitative component, small subgroup sizes, and the primary goal of supporting mixed-methods integration rather than confirmatory testing, we chose to emphasize descriptive median/IQR patterns and integrated interpretation rather than introduce effect-size estimates that may be unstable in small subgroups.

7. The use of domain-level scores created by averaging Likert items should be justified. Because Likert responses are ordinal and the instrument is newly developed, the authors should explain why averaging items was appropriate and whether sensitivity analyses were performed using item-level responses only. If domain-level scores are not central to the interpretation, the authors may consider simplifying the analysis and focusing primarily on item-level descriptive findings.

Response:

We thank the reviewer for this important clarification. We agree that domain-level scores from averaged Likert items require cautious interpretation, given the newly developed instrument and ordinal response scale.

Quantitative measures and analysis (p.11, lines 248–254): “Because the questionnaire was newly developed and the Likert responses were ordinal, the primary quantitative interpretation was conducted at the item level. Participant-level domain summaries were calculated by averaging responses to conceptually related items within each predefined domain; medians and IQRs of these summaries were reported descriptively. Domain summaries were not used to select items for the joint displays or to derive the principal integrated findings.”

The main joint display tables (Tables 3–5) were based on item-level median/IQR patterns and qualitative relevance rather than domain-level averages, as clarified in the Mixed-methods integration and selection of joint display items section (p.13, lines 294–301).

S3 File now provides complete item-level results alongside an explicit statement of the descriptive role of domain scores: “This supplementary material provides the complete item-level quantitative results from the provider questionnaires... Domain-level summaries are provided for descriptive overview only... Because Likert-scale responses are ordinal and the questionnaire was newly developed, the primary interpretation of quantitative findings was conducted at the item level. Domain-level scores were not used to select items for the main joint displays or to support confirmatory inference.”

Since the primary analysis and integrated interpretation were already item-level rather than domain-based, a separate sensitivity analysis was not required for inferential purposes; the complete item-level results in S3 File nonetheless allow readers to compare item-level and domain-level patterns for full transparency. These revisions clarify that domain-level scores are supplementary descriptive summaries, not central analytic outcomes.

8. The joint displays are useful, but the selection process may create concern about selective reporting. The manuscript states that Tables 3-5 include selected items based on predefined analytic criteria rather than all measured items. This approach can be acceptable in mixed-methods research, but the authors should ensure that the main manuscript provides sufficient information for readers to judge whether the selected items fairly represent the full dataset. The complete item-level results should be clearly provided in the supplementary materials, and the manuscript should explicitly state that the joint displays are illustrative rather than exhaustive.

Response:

We thank the reviewer for this important recommendation. We agree the selection process for the joint displays must be presented transparently to avoid concerns about selective reporting.

Mixed-methods integration and selection of joint display items (p.13, lines 294–301) now reports the predefined criteria: “Quantitative items were selected from the complete questionnaire dataset using predefined analytic criteria. Table 3 included barrier items with median ratings ≥4.0 in at least two professional groups and relevant qualitative material; comorbidity affecting clinical suitability was retained as a prespecified analytic exception because it was prominent in the qualitative responses. Table 4 included the highest-rated profession-specific obstacle for each provider group. Table 5 included supportive factors with median ratings ≥4.0 across all three groups and relevant qualitative suggestions.”

The manuscript also states the displays are illustrative, not exhaustive (p.13, lines 302–306): “The joint displays are illustrative rather than exhaustive and were not intended to rank barriers or supportive factors by prevalence or importance. Qualitative excerpts were used to show how participants described the selected items and were not treated as frequency estimates. Complete item-level results, p-values, and the rationale for item selection are provided in S3 File.”

S3 File now provides complete item-level results alongside the selection rationale: “This supplementary material provides the complete item-level quantitative results from the provider questionnaires and explains the analytic rationale for selecting items presented in the main manuscript joint displays (Tables 3–5). Items were selected to illustrate analytically relevant patterns for mixed-methods integration, rather than to exhaustively represent or rank all measured questionnaire items. Qualitative excerpts in the joint displays were used to contextualize quantitative patterns and were not intended to indicate prevalence, relative importance, or statistical weighting. Providing the full quantitative results alongside the selection rationale enhances transparency and interpretability of the integrated findings.”

Together, these revisions make the selection process transparent and let readers assess the selected items against the complete dataset.

9. The discussion and conceptual interpretation should be shortened and made more cautious. Terms such as “operational misalignment,” “workflow execution,” “role-specific expectations,” and “system-level operationalization” are repeated frequently. These concepts may be useful, but the study did not directly observe workflows, referral processes, reimbursement pathways, surgical scheduling, patient behavior, or actual AV access outcomes. The data primarily reflect provider perceptions. Therefore, the authors should revise the Discussion to distinguish clearly between observed survey findings, qualitative interpretations, and hypotheses about system-level mechanisms.

Response:

We thank the reviewer for this important comment. We agree that terms implying observed mechanisms, workflows, or operational processes were not appropriate, given that this study captured provider-reported perceptions rather than directly observed workflows, referral processes, reimbursement pathways, surgical scheduling, patient behavior, or AV access outcomes.

We revised the Discussion throughout to remove such language and consistently distinguish observed survey findings from qualitative interpretation and hypothesis-generating inference. Terms such as “operational misalignment,” “workflow execution,” “role-specific expectations,” and “system-level operationalization” have been removed. The Discussion now consistently uses provider-perception framing, for example:

“These findings represent provider-reported, hypothesis-generating insights intended to inform future service evaluation.” (p.20, lines 449–450)

“These are descriptive because the items differed across professions, subgroup sizes were uneven, and the three sets of role-specific items were not directly compared statistically.” (p.22, lines 496–498)

“The patterns do not necessarily indicate disagreement across professions; rather, they show how shared priorities may be understood through different professional lenses. This interpretation generates testable questions for future studies...” (p.23, lines 528–533)

These revisions ensure the Discussion clearly separates what was directly measured (provider ratings and brief written responses) from interpretive and hypothesis-generating statements about underlying mechanisms.

10. The manuscript would also benefit from clearer acknowledgment that objective clinical and system-level outcome data were not collected. The central issue is timely predialysis AV access planning and creation, but the study does not measure actual AVF/AVG creation rates, referral-to-surgery time, catheter use at dialysis initiation, failed referrals, surgical wait times, reimbursement delays, or patient-level reasons for noncompletion. This does not invalidate the study, but it limits what can be concluded. The authors should present their findings as perceived barriers and facilitators rather than determinants of actual delayed access creation.

Response:

We thank the reviewer for this clarification. We agree the study should not be interpreted as identifying determinants of actual delayed AV access creation, since objective clinical and system-level outcome data were not collected. We revised the manuscript throughout to present findings as provider-reported perceptions rather than causal or objective determinants of delayed access.

Table 1 note (p.15, lines 337–338): “These provider-estimated proportions of predialysis AV access were self-reported by providers and are presented as contextual practice estimates, not audited institutional rates.”

Strengths and limitations (p.28, lines 654–665): “Finally, the study did not include objective measures of catheter use at dialysis initiation, audited AVF or AVG creation rates, referral completion, referral-to-procedure intervals, failed referrals, procedural waiting time, reimbursement delays, or patient-level reasons for noncompletion. Provider-estimated predialysis access percentages were contextual estimates rather than audited institutional rates... Accordingly, the findings identify provider-perceived barriers and priorities for future evaluation rather than determinants of actual delayed access creation. Future studies should integrate provider and patient perspectives with clinical records, referral and scheduling data, reimbursement information, and access status at hemodialysis initiation.”

Conclusion (p.28, lines 667–677): “In this exploratory regional provider-perception study, healthcare providers in Upper Northern Thailand identified patient-, clinical-, care pathway, system-, and policy-level barriers to timely predialysis AV access preparation... These preliminary, context-specific findings may inform locally adapted service improvement and the design of future evaluations. Further studies integrating provider and patient perspectives with clinical and care-pathway data are needed to assess the feasibility and effect of the proposed strategies on timely AV access preparation and access status at hemodialysis initiation.”

These revisions clarify the inferential boundaries of the study while preserving its contribution as a mixed-methods analysis of multidisciplinary provider perspectives.

11. The title, abstract, and conclusion should be revised to avoid overgeneralization. Because the study was conducted in Upper Northern Thailand, the authors should avoid phrasing that implies national representativeness. The abstract should clearly state the regional setting and exploratory nature of the study. The conclusion should emphasize that the findings provide preliminary, context-specific insights that may inform future service improvement work, rather than implying that the proposed strategies have been proven effective.

Response:

We thank the reviewer for this recommendation. We revised the title, abstract, and conclusion to avoid overgeneralization and clearly present the study as exploratory and regional to Upper Northern Thailand.

Title (p.1, lines 1–4): “Multidisciplinary Providers' Perspectives on Timely Arteriovenous Access Planning Prior to Hemodialysis: An Exploratory Mixed-Methods Study in Upper Northern Thailand”

Abstract Background (p.3, lines 58–61): “This exploratory regional study compared the perspectives of nephrologists, vascular surgeons, and dialysis nurses in Upper Northern Thailand and identified supportive strategies that providers considered relevant to improving timely AV access preparation.”

Abstract Methods (p.3, lines 63–66): “We conducted a cross-sectional convergent mixed-methods study using a self-administered survey among nephrologists, vascular surgeons, and dialysis nurses practicing in hospitals and dialysis clinics across Upper Northern Thailand from February to April 2025.”

Abstract Conclusions (p.4, lines 84–88): “Providers in this regional study identified shared priorities for improving timely predialysis AV access preparation, while differing in the meanings and considerations they associated with those priorities. These context-specific, hypothesis-generating findings may inform locally adapted service improvement and future evaluation of the proposed strategies.”

Conclusion (p.28, lines 667–677): “In this exploratory regional provider-perception study, healthcare providers in Upper Northern Thailand identified patient-, clinical-, care pathway, system-, and policy-level barriers to timely predialysis AV access preparation. Across professional groups, providers shared several priority barriers and supportive strategies, while emphasizing different role-related considerations. These preliminary, context-specific findings may inform locally adapted service improvement and the design of future evaluations. Further studies integrating provider and patient perspectives with clinical and care-pathway data are needed to assess the feasibility and effect of the proposed strategies on timely AV access preparation and access status at hemodialysis initiation.”

These revisions clarify that the study provides preliminary, context-specific insights from Upper Northern Thailand and does not claim national representativeness or proven effectiveness of the proposed strategies.

12. The writing is generally clear, but the manuscript is repetitive and could be substantially shortened. Phrases such as “predialysis AV access planning and creation,” “system-level strategies,” “role-specific interpretations,” “workflow constraints,” and “operational misalignment” recur throughout the manuscript. The Discussion in particular would benefit from consolidation. The authors should reduce repeated conceptual language and more directly link each interpretation to specific findings.

Response:

We thank the reviewer for this observation. We revised the manuscript to reduce repeated conceptual phrasing and consolidate the Discussion, linking interpretations more directly to specific findings. Repeated phrases — “system-level strategies,” “role-specific interpretations,” “workflow constraints,” “operational misalignment” — were removed or replaced with findings-specific language. The Discussion subsections were consolidated to link directly to the corresponding items in Tables 3–5, reducing conceptual repetition.

13. Finally, the reference list should be carefully checked before publication. Most references appear relevant, but several entries may require correction of author formatting, publication year, article type, DOI, or bibliographic details. The authors should verify all references against PubMed, Crossref, ThaiJO, and publisher websites, and add DOI information where available.

Response:

We thank the reviewer for this comment. We have carefully checked the reference list against PubMed, Crossref, ThaiJO, and publisher websites, and corrected author formatting, publication years, article types, and bibliographic details as needed. DOI information has been added where available.

Attachment Submitted filename: Response to Reviewers_R1.docx

# Peer review: Decision Letter 1

**DOI:** 10.1371/journal.pone.0359863.r003

**Authors:** Jeerath Phannajit

2 Sep 2026

Dear Dr. Rerkasem,

Thank you for submitting your manuscript to PLOS One. After careful consideration, we feel that it has merit but does not fully meet PLOS One’s publication criteria as it currently stands. Therefore, we invite you to submit a revised version of the manuscript that addresses the points raised during the review process.

Please submit your revised manuscript by Oct 17 2026 11:59PM. If you will need more time than this to complete your revisions, please reply to this message or contact the journal office at plosone@plos.org. When you're ready to submit your revision, log on to https://www.editorialmanager.com/pone/ and select the 'Submissions Needing Revision' folder to locate your manuscript file.

- A letter that responds to each point raised by the academic editor and reviewer(s). You should upload this letter as a separate file labeled 'Response to Reviewers'.
- A marked-up copy of your manuscript that highlights changes made to the original version. You should upload this as a separate file labeled 'Revised Manuscript with Track Changes'.
- An unmarked version of your revised paper without tracked changes. You should upload this as a separate file labeled 'Manuscript'.

If you would like to make changes to your financial disclosure, please include your updated statement in your cover letter. Guidelines for resubmitting your figure files are available below the reviewer comments at the end of this letter.

If applicable, we recommend that you deposit your laboratory protocols in protocols.io to enhance the reproducibility of your results. Protocols.io assigns your protocol its own identifier (DOI) so that it can be cited independently in the future. For instructions see: https://journals.plos.org/plosone/s/submission-guidelines#loc-laboratory-protocols. Additionally, PLOS One offers an option for publishing peer-reviewed Lab Protocol articles, which describe protocols hosted on protocols.io. Read more information on sharing protocols at https://plos.org/protocols?utm_medium=editorial-email&utm_source=authorletters&utm_campaign=protocols.

As the corresponding author, your ORCID iD is verified in the submission system and will appear in the published article. PLOS supports the use of ORCID, and we encourage all coauthors to register for an ORCID iD and use it as well. Please encourage your coauthors to verify their ORCID iD within the submission system before final acceptance, as unverified ORCID iDs will not appear in the published article. *Only*  the individual author can complete the verification step; PLOS staff *cannot*  verify ORCID iDs on behalf of authors.

We look forward to receiving your revised manuscript.

Kind regards,

Jeerath Phannajit, M.D, Ph.D.

Academic Editor

PLOS One

Journal Requirements:

If the reviewer comments include a recommendation to cite specific previously published works, please review and evaluate these publications to determine whether they are relevant and should be cited. There is no requirement to cite these works unless the editor has indicated otherwise.

Please review your reference list to ensure that it is complete and correct. If you have cited papers that have been retracted, please include the rationale for doing so in the manuscript text, or remove these references and replace them with relevant current references. Any changes to the reference list should be mentioned in the rebuttal letter that accompanies your revised manuscript. If you need to cite a retracted article, indicate the article’s retracted status in the References list and also include a citation and full reference for the retraction notice.

Additional Editor Comments:

Please carefully recheck the grammatical errors and terms consistency as stated by reviewer 2.

[Note: HTML markup is below. Please do not edit.]

Reviewers' comments:

Reviewer's Responses to Questions

**Comments to the Author**

Reviewer #1: All comments have been addressed

Reviewer #2: All comments have been addressed

**********

2. Is the manuscript technically sound, and do the data support the conclusions?

Reviewer #1: Yes

Reviewer #2: Yes

**********

3. Has the statistical analysis been performed appropriately and rigorously?

Reviewer #1: Yes

Reviewer #2: Yes

**********

4. Have the authors made all data underlying the findings in their manuscript fully available?

The PLOS Data policy

Reviewer #1: Yes

Reviewer #2: Yes

**********

5. Is the manuscript presented in an intelligible fashion and written in standard English?

Reviewer #1: Yes

Reviewer #2: Yes

**********

Reviewer #1: Thank you the authors for your thorough and thoughtful revisions. The authors have addressed all of my comments clearly and incorporated the necessary modifications into the manuscript. I am fully satisfied with the responses.

Reviewer #2: The authors have substantially improved the manuscript and have responded carefully to the major methodological and interpretative concerns raised during the previous review. In particular, the revised manuscript now appropriately presents the study as an exploratory, regional, provider-perception study; provides a clearer description of the sampling strategy and questionnaire development; acknowledges the predominance of dialysis nurses and the absence of a separately sampled CKD/predialysis nursing group; and appropriately distinguishes provider-reported perceptions from objective clinical or health-system outcomes. The conclusions are also considerably more cautious and better aligned with the study design. I do not believe that further major methodological revision or additional analysis is required.

Before the manuscript can be accepted, I suggest that the authors address the following minor editorial and consistency issues:

1. Please undertake a careful final English-language proofread of the entire manuscript. Several grammatical and wording errors remain, including subject–verb agreement, pluralization, duplicated wording, and typographical errors. Examples include “AV access preparation remain suboptimal,” “Integrated finding identified,” “in many setting,” “For supportive factors received,” “these ratting patterns,” and “Across professional groups, provider shared.” These issues do not affect the scientific conclusions but should be corrected to improve clarity and readability.

2. Please correct the incomplete sentence in the Results section describing insurance and reimbursement barriers. The current text ends with “qualitative responses referring to coverage limitations and administrative” before immediately proceeding to the sentence concerning comorbidity and clinical suitability. The missing wording should be restored so that the paragraph is grammatically complete and its intended meaning is clear.

3. Please perform a final consistency check of terminology throughout the manuscript. Terms such as “predialysis AV access preparation,” “planning and creation,” “supportive factors,” “supportive strategies,” and “facilitators” are sometimes used interchangeably. Complete uniformity is not necessary where the concepts genuinely differ, but terminology should be used consistently within the Abstract, Results, Discussion, tables, figure, and Conclusion.

4. Please carefully recheck the reference list against the original publications. There appear to be remaining bibliographic and formatting inconsistencies, including misplaced or potentially incorrect DOI information and irregular formatting of some references. For example, the entries for Xi et al., Allon et al., and Sachdeva and Abreo should be verified carefully. Journal names, punctuation, author formatting, article titles, volume/page information, and DOI assignments should all be checked before final acceptance.

5. Please conduct one final editorial check of tables, Supporting Information citations, and figure references. The revised manuscript has substantially improved the transparency of the joint-display selection criteria and the distinction between quantitative findings and qualitative contextualization. The authors should ensure that references to S1–S4 Files, Tables 1–5, and Figure 1 are internally consistent and that all captions and notes use the final terminology adopted in the main text.

**********

what does this mean?). If published, this will include your full peer review and any attached files.

If you choose “no”, your identity will remain anonymous but your review may still be made public.

**Do you want your identity to be public for this peer review?**  For information about this choice, including consent withdrawal, please see our Privacy Policy

Reviewer #1: No

Reviewer #2: **Yes:**  Wisit Kaewput, MD, FRCP(London), FRCP(Glasgow), FASN, MFRCP(Thailand), FAcadMEd

**********

[NOTE: If reviewer comments were submitted as an attachment file, they will be attached to this email and accessible via the submission site. Please log into your account, locate the manuscript record, and check for the action link "View Attachments". If this link does not appear, there are no attachment files.]

To ensure your figures meet our technical requirements, please review our figure guidelines: https://journals.plos.org/plosone/s/figures

You may also use PLOS’s free figure tool, NAAS, to help you prepare publication quality figures: https://journals.plos.org/plosone/s/figures#loc-tools-for-figure-preparation.

NAAS will assess whether your figures meet our technical requirements by comparing each figure against our figure specifications.

# Author comment: Author response to Decision Letter 2

**DOI:** 10.1371/journal.pone.0359863.r004

9 Sep 2026

Second revision

PONE-D-26-17428R1

Response to editor and reviewers

Journal: PLOS ONE

Manuscript ID: PONE-D-26-17428R1

Title: Multidisciplinary Providers’ Perspectives on Timely Arteriovenous Access Planning Prior to Hemodialysis: An Exploratory Mixed-Methods Study in Upper Northern Thailand

Corresponding author: Kittipan Rerkasem, M.D.

Date: 9 Sep 2026

Dear Dr. Phannajit and Reviewers,

Thank you for the careful evaluation of our revised manuscript and for the constructive comments. We have addressed each point raised by the Academic Editor and Reviewer #2 and have carefully checked the manuscript for language, terminology, references, and internal consistency. Reviewer comments are reproduced below, followed by our point-by-point responses. Changes are shown in the marked-up manuscript and incorporated into the clean revised manuscript.

We hope that the revised manuscript is now suitable for publication in PLOS ONE.

Sincerely,

Kittipan Rerkasem, M.D.

On behalf of all authors

Response to the Academic Editor

Comment: Please carefully recheck the grammatical errors and terms consistency as stated by reviewer 2.

Response: Thank you for this guidance. We carefully proofread the entire manuscript and corrected the remaining grammatical, typographical, and wording errors. We also performed a final terminology consistency check across the main text, tables, Figure 1, and S1–S4 Files. The terminology is now used consistently according to the distinctions described in our responses to Reviewer #2. All revisions are shown in the marked-up files and do not alter the study findings.

Response to Reviewer #1

Comment: Thank you to the authors for your thorough and thoughtful revisions. The authors have addressed all of my comments clearly and incorporated the necessary modifications into the manuscript. I am fully satisfied with the responses.

Response: We sincerely thank the reviewer for the careful evaluation of our revised manuscript and for confirming that the previous comments have been adequately addressed. We greatly appreciate the reviewer’s constructive feedback, which has helped us improve the clarity and quality of the manuscript.

Response to Reviewer #2

We sincerely thank Reviewer #2 for the careful reassessment of our manuscript and for recognizing the substantive improvements made during the previous revision. We have addressed each remaining editorial and consistency issue below.

Comment 1. Please undertake a careful final English-language proofread of the entire manuscript. Several grammatical and wording errors remain, including subject–verb agreement, pluralization, duplicated wording, and typographical errors. Examples include “AV access preparation remain suboptimal,” “Integrated finding identified,” “in many setting,” “For supportive factors received,” “these ratting patterns,” and “Across professional groups, provider shared.” These issues do not affect the scientific conclusions but should be corrected to improve clarity and readability.

Response: Thank you for identifying these language and wording issues. We carefully proofread the entire manuscript and corrected errors in subject–verb agreement, pluralization, sentence construction, word choice, spelling, punctuation, duplicated wording, and typographical presentation. Specifically, “AV access preparation remain suboptimal” was corrected to “AV access preparation remains suboptimal”; “Integrated finding identified” was revised to “Integration of the quantitative and qualitative findings identified”; “in many setting” was corrected to “in many settings”; “For supportive factors received” was corrected to “Four supportive factors received”; “these ratting patterns” was corrected to “these rating patterns”; and “Across professional groups, provider shared” was corrected to “Across professional groups, providers shared.”

We also corrected additional language and wording issues throughout the manuscript to improve clarity, readability, and consistency. These editorial revisions did not alter the study data, analyses, results, or interpretation.

Changes in the manuscript: Corrections were made throughout the Abstract, Introduction, Methods, Results, Discussion, Conclusion, tables, and Figure 1 caption and are shown in the marked-up manuscript.

Comment 2. Please correct the incomplete sentence in the Results section describing insurance and reimbursement barriers. The current text ends with “qualitative responses referring to coverage limitations and administrative” before immediately proceeding to the sentence concerning comorbidity and clinical suitability. The missing wording should be restored so that the paragraph is grammatically complete and its intended meaning is clear.

Response: Thank you for identifying this incomplete sentence. The final portion of the sentence was inadvertently omitted during the previous revision. We have restored the missing wording and corrected the duplicated expression “barriers constraints.” The revised sentence now reads: “Insurance coverage and reimbursement processes received median ratings of 4.0 among nurses and surgeons and 3.5 among nephrologists, with qualitative responses referring to coverage limitations and administrative delays.” The paragraph is now grammatically complete and clearly conveys the intended finding.

Changes in the manuscript: Results, subsection “Integrated barriers to timely predialysis AV access preparation,” page 16, lines 436–438.

Comment 3. Please perform a final consistency check of terminology throughout the manuscript. Terms such as “predialysis AV access preparation,” “planning and creation,” “supportive factors,” “supportive strategies,” and “facilitators” are sometimes used interchangeably. Complete uniformity is not necessary where the concepts genuinely differ, but terminology should be used consistently within the Abstract, Results, Discussion, tables, figure, and Conclusion.

Response: Thank you for this helpful comment. We conducted a final terminology review across the Abstract, main text, tables, Figure 1, Conclusion, and Supporting Information. We now use “predialysis AV access preparation” as the umbrella term encompassing planning and, when clinically appropriate, surgical creation of an AVF or AVG before hemodialysis initiation. “Planning and creation” is retained only where the text specifically refers to these distinct stages of preparation.

We use “supportive factors” for the six quantitative questionnaire items, “supportive strategies” when discussing the four consistently highly rated factors as candidate components for future service evaluation, and “provider-suggested actions” for recommendations derived from the open-ended responses. “Facilitators” is no longer used as an analytic label in the main manuscript and is retained only in S1 File, where it reproduces the wording of the original open-ended questionnaire item.

Relevant headings, table labels, captions, notes, Figure 1, and Supporting Information files were revised accordingly. These changes improve terminological consistency without altering the study data, analyses, or findings.

Changes in the manuscript: Terminology was standardized throughout the Abstract, Introduction, Methods, Results, Discussion, Conclusion, Tables 1–5, Figure 1 and its caption, and S1–S4 Files, as shown in the marked-up files.

Comment 4. Please carefully recheck the reference list against the original publications. There appear to be remaining bibliographic and formatting inconsistencies, including misplaced or potentially incorrect DOI information and irregular formatting of some references. For example, the entries for Xi et al., Allon et al., and Sachdeva and Abreo should be verified carefully. Journal names, punctuation, author formatting, article titles, volume/page information, and DOI assignments should all be checked before final acceptance.

Response: Thank you for drawing our attention to these bibliographic inconsistencies. We carefully rechecked the entire reference list against the original publications and corrected the journal names, punctuation, author formatting, article titles, publication years, volume and page information, and DOI assignments, as appropriate.

In particular, the DOI for Xi et al., which had been inadvertently placed under the Allon et al. entry, was reassigned to the correct reference. The Allon et al. entry was updated with its correct DOI and standardized bibliographic information. The Sachdeva and Abreo entry was also corrected to remove duplicated journal information and irregular punctuation and to standardize the author and article-title formatting. Additional inconsistencies elsewhere in the reference list were corrected to ensure a uniform bibliographic format.

Comment 5. Please conduct one final editorial check of tables, Supporting Information citations, and figure references. The revised manuscript has substantially improved the transparency of the joint-display selection criteria and the distinction between quantitative findings and qualitative contextualization. The authors should ensure that references to S1–S4 Files, Tables 1–5, and Figure 1 are internally consistent and that all captions and notes use the final terminology adopted in the main text.

Response: Thank you for this helpful comment. We conducted a final consistency check of Tables 1–5, Figure 1, and S1–S4 Files. All citations, captions, labels, and notes were corrected and harmonized with the terminology used in the main text. Quantitative findings, qualitative contextualization, and provider-suggested actions are now clearly distinguished throughout.

Changes in the manuscript and Supporting Information: Relevant cross-references and terminology were standardized throughout the marked-up manuscript, Figure 1, and S1–S4 Files.

Attachment Submitted filename: Response_to_Editor_and_Reviewers_R2.docx

# Editor report: Decision Letter 2

**DOI:** 10.1371/journal.pone.0359863.r005

**Authors:** Jeerath Phannajit

20 Sep 2026

Multidisciplinary Providers’ Perspectives on Timely Arteriovenous Access Planning Prior to Hemodialysis: An Exploratory Mixed-Methods Study in Upper Northern Thailand

PONE-D-26-17428R2

Dear Dr. Rerkasem,

We’re pleased to inform you that your manuscript has been judged scientifically suitable for publication and will be formally accepted for publication once it meets all outstanding technical requirements.

Within one week, you’ll receive an e-mail detailing the required amendments. When these have been addressed, you’ll receive a formal acceptance letter and your manuscript will be scheduled for publication.

An invoice will be generated when your article is formally accepted. Please note, if your institution has a publishing partnership with PLOS and your article meets the relevant criteria, all or part of your publication costs will be covered. Please make sure your user information is up-to-date by logging into Editorial Manager at Editorial Manager® and clicking the ‘Update My Information' link at the top of the page. For questions related to billing, please contact billing support.

If your institution or institutions have a press office, please notify them about your upcoming paper to help maximize its impact. If they’ll be preparing press materials, please inform our press team as soon as possible -- no later than 48 hours after receiving the formal acceptance. Your manuscript will remain under strict press embargo until 2 pm Eastern Time on the date of publication. For more information, please contact onepress@plos.org.

Kind regards,

Jeerath Phannajit, M.D, Ph.D.

Academic Editor

PLOS One

Additional Editor Comments (optional):

Reviewers' comments:

# Editor report: Acceptance letter

**DOI:** 10.1371/journal.pone.0359863.r006

**Authors:** Jeerath Phannajit

PONE-D-26-17428R2

PLOS One

Dear Dr. Rerkasem,

I'm pleased to inform you that your manuscript has been deemed suitable for publication in PLOS One. Congratulations! Your manuscript is now being handed over to our production team.

At this stage, our production department will prepare your paper for publication. This includes ensuring the following:

* All references, tables, and figures are properly cited

* All relevant supporting information is included in the manuscript submission,

* There are no issues that prevent the paper from being properly typeset

You will receive further instructions from the production team, including instructions on how to review your proof when it is ready. Please keep in mind that we are working through a large volume of accepted articles, so please give us a few days to review your paper and let you know the next and final steps.

Lastly, if your institution or institutions have a press office, please let them know about your upcoming paper now to help maximize its impact. If they'll be preparing press materials, please inform our press team within the next 48 hours. Your manuscript will remain under strict press embargo until 2 pm Eastern Time on the date of publication. For more information, please contact onepress@plos.org.

You will receive an invoice from PLOS for your publication fee after your manuscript has reached the completed accept phase. If you receive an email requesting payment before acceptance or for any other service, this may be a phishing scheme. Learn how to identify phishing emails and protect your accounts at https://explore.plos.org/phishing.

If we can help with anything else, please email us at customercare@plos.org.

Thank you for submitting your work to PLOS One and supporting open access.

Kind regards,

PLOS One Editorial Office Staff

on behalf of

Dr. Jeerath Phannajit

Academic Editor

PLOS One
